# The Reassessed Potential of SARS-CoV-2 Attenuation for COVID-19 Vaccine Development—A Systematic Review

**DOI:** 10.3390/v14050991

**Published:** 2022-05-07

**Authors:** Marcin Goławski, Piotr Lewandowski, Iwona Jabłońska, Marcin Delijewski

**Affiliations:** 1 Department of Pharmacology, Faculty of Medical Sciences in Zabrze, Medical University of Silesia, 41-808 Katowice, Poland; lewandop@icloud.com (P.L.); mdelijewski@sum.edu.pl (M.D.); 2 Department of Biophysics, Faculty of Medical Sciences in Zabrze, Medical University of Silesia, 41-808 Katowice, Poland; jablonska.iwo@gmail.com

**Keywords:** coronavirus, SARS-CoV-2, COVID-19, live attenuated vaccine, review

## Abstract

Live-attenuated SARS-CoV-2 vaccines received relatively little attention during the COVID-19 pandemic. Despite this, several methods of obtaining attenuated coronaviruses are known. In this systematic review, the strategies of coronavirus attenuation, which may potentially be applied to SARS-CoV-2, were identified. PubMed, Scopus, Web of Science and Embase databases were searched to identify relevant articles describing attenuating mutations tested in vivo. In case of coronaviruses other than SARS-CoV-2, sequence alignment was used to exclude attenuating mutations that cannot be applied to SARS-CoV-2. Potential immunogenicity, safety and efficacy of the attenuated SARS-CoV-2 vaccine were discussed based on animal studies data. A total of 27 attenuation strategies, used to create 101 different coronaviruses, have been described in 56 eligible articles. The disruption of the furin cleavage site in the SARS-CoV-2 spike protein was identified as the most promising strategy. The replacement of core sequences of transcriptional regulatory signals, which prevents recombination with wild-type viruses, also appears particularly advantageous. Other important attenuating mutations encompassed mostly the prevention of evasion of innate immunity. Sufficiently attenuated coronaviruses typically caused no meaningful disease in susceptible animals and protected them from challenges with virulent virus. This indicates that attenuated COVID-19 vaccines may be considered as a potential strategy to fight the threat posed by SARS-CoV-2.

## 1. Introduction

The COVID-19 pandemic continues to be a pressing global public health problem [1]. Despite the extreme amount of effort put into the development of vaccines and drug repurposing [2,3], the potential of attenuated SARS-CoV-2 as a vaccine has received comparatively little attention, with only two candidates in clinical or pre-clinical trials [4,5].

SARS-CoV-2 is an etiologic factor of COVID-19. As of 10 April 2022, 496,507,539 cases have been diagnosed globally, and the disease led to 6,177,354 deaths [1]. SARS-CoV-2 primarily affects the upper and lower respiratory tract, it can cause severe infection and may lead to death [6,7]. The symptoms are typical of any viral respiratory infection, although loss of taste or smell is notable and gastrointestinal symptoms may also be present [8,9]. The virus may also be present in several other tissues and organs, including heart and brain [6,10]. This may explain several cardiovascular and neurological complications observed during the course of COVID-19 [10,11].

Vaccine research and development in the case of SARS-CoV-2 and COVID-19 is focused on the spike (S) protein because it is a target of neutralizing antibodies. Its receptor binding domain (RBD) has the highest concentration of neutralizing epitopes, and some vaccines contain the RBD instead of a full S protein [12,13]. Indeed, serum neutralizing antibodies, as well as anti-Spike and anti-RBD humoral responses, appear to be correlated with vaccine efficacy and protection based on the data from human trials of commercially marketed vaccines [14,15,16,17]. The exact importance of cellular immunity is less understood [18,19]. Nevertheless, some animal studies have shown the nucleocapsid protein and its peptides to elicit a somewhat effective T cell-mediated immune response against SARS-CoV-2 [20,21,22].

Attenuated viral vaccines are often regarded as somewhat unsafe due to attenuated virus’ ability to spread, mutate and recombine with wild-type (WT) strains [23]. Nevertheless, several live attenuated vaccines (LAVs) are included in the WHO List of Essential Medicines and therefore still seem to be a valid way to combat infectious diseases [24]. This view is also supported by the existence of attenuated smallpox vaccines based on Modified Vaccinia Ankara (MVA), which can be administered to immunocompromised individuals. Indeed, MVA lacks the ability to replicate in mammalian cells [25]. LAVs may pose several advantages in the context of SARS-CoV-2 and COVID-19, such as rapid development through the use of reverse genetics, elicitation of both mucosal and systemic immunity of cellular as well as humoral type, the widest array of antigens presented, needle-free administration, high production efficiency and high efficacy [26,27].

Attenuated coronaviruses are a subject of a relatively large volume of research. Moreover, vaccines against several veterinary coronaviruses were commercialized, including infectious bronchitis virus (IBV) [28], which affects chickens; porcine epidemic diarrhea (PEDV) and transmissible gastroenteritis virus (TGEV), which affect pigs [29,30]; feline infectious peritonitis virus (FIPV) [31]; canine coronavirus (CCoV) [30] and bovine coronavirus (BCoV) [32]. Some other coronaviruses, including murine hepatitis virus (MHV) [33], severe acute respiratory syndrome coronavirus (SARS-CoV) [34], middle east respiratory coronavirus (MERS-CoV) [35] and—most recently—SARS-CoV-2 [36] were successfully attenuated and studied in vivo. Attenuated IBV vaccines are of significant importance. Many vaccines are available due to the existence of different strains and limited cross-protection [28,37]. On the other hand, attenuated IBV viruses can recombine to yield new strains [38,39,40,41], while the sequences of vaccine strains themselves are frequently not known [42]. Nevertheless, attenuated IBV vaccines are routinely used and efficacious [43,44]. Attenuated PEDV vaccines are primarily used in Asia, but, unfortunately, the emergence of new strains has reduced their usefulness [45,46]. Some attenuated coronavirus vaccines—such as ones against PEDV and TGEV—are administered to pregnant animals to provide lactogenic immunity to the offspring [29,30]. Attenuated IBV vaccines are widely used and applied as coarse-spray, which allows for the easy mass-vaccination of chicks [28].

Attenuated coronaviruses may be obtained through the old approach, which is serial passage in cell culture [30]. IBV can also be passaged in embryonated chicken eggs, and this is how the commercially available vaccines have been obtained [28]. This process yields random results by design but may be influenced by the addition of antiviral agents or antibodies to obtain escape mutants. Virus behavior at different temperatures may be influenced by altered passage temperature. Cold-adapted viruses grow to higher titers at lower temperatures, while temperature-sensitive viruses fail to replicate efficiently at high temperatures [47,48,49]. The accumulation of random mutations during passages may be accelerated with mutagens [47]. On the other hand, the precise mechanisms of attenuation that result after several passages in cell culture do not appear to be precisely determined.

On rare occasions, an attenuated strain of a coronavirus may occur naturally. A good example is the porcine respiratory coronavirus (PRCV), which is a deletion mutant of TGEV with altered tropism. It usually causes a mild respiratory disease instead of a lethal enteric one [50,51,52].

A newer approach is to create the recombinant attenuated coronavirus with specific mutations through the use of reverse genetics systems [53]. Coronaviruses have some of the largest genomes of all known RNA viruses [54], which, along with the presence of sequences toxic to *Escherichia coli* [55,56], makes manipulation of their genomes somewhat difficult [53]. Methods of assembling large coronavirus genomes include but are not limited to restriction-ligation [56,57,58], in-fusion cloning [59], circular polymerase extension reaction [60] or assembly in yeast [61]. Coronaviral cDNA may be cloned into plasmids or bacterial artificial chromosomes or recombined within the vaccinia virus [53,62]. It may be transfected into mammalian cells and transcribed by endogenous RNA polymerase to yield functional and replicating coronavirus genomes. Instead of intracellular transcription, in vitro transcription and transfection with RNA may also be utilized [53]. It also is possible to recombine an RNA fragment with the coronavirus in cells [53]. However, most of these techniques do not differ significantly in major limitations and final results [53].

Reverse genetics allows for the creation of coronaviruses that could not be obtained during serial passage in cell culture due to reduced fitness, which precludes them from appearing in culture in significant proportion. Some chimeric coronaviruses [63] and coronaviruses that encode genes with reporter and other functions [64] cannot be obtained in any other way. Since numerous proteins and functions are conserved between coronavirus species, it is possible to create the same mutations in different coronaviruses [49,65,66,67].

SARS-CoV-2 emerged in 2019 in Wuhan, China [68] and is one of the major challenges for contemporary virology, pharmacology and vaccinology. Despite this, for many coronavirus-attenuating mutations that are known, no reports of successful implementation in SARS-CoV-2 have been published. For example, non-structural protein 16 (NSP16) is responsible for the 2′-O-methylation of coronaviral RNA cap [69], and several active site—or binding site—mutations that reduce or disable its activity have already been applied to MHV, PEDV, SARS-CoV and MERS-CoV. The resulting mutant viruses have been shown to possess an attenuated phenotype in vivo [34,70,71,72].

This systematic review aimed to identify mutations that have been applied to—or could be applied to—SARS-CoV-2 to attenuate it based on a literature search. The obtained results were analyzed to determine the potential features of attenuated SARS-CoV-2 vaccines. Particular focus was paid to the efficacy and safety of such vaccines. In order to increase the quality of the reported evidence, and to reasonably limit the number of research papers included in this review, while simultaneously taking into account the absence of the published results from trials involving humans, the analyzed studies were limited to those presenting in vivo data on attenuated COVID-19 vaccines from animal models. It was concluded that the furin cleavage site (FCS) deletion is the most promising strategy, although it perhaps needs to be used in combination. It was also determined that a sufficiently attenuated SARS-CoV-2 virus is unlikely to cause any significant pathology but is likely to be sufficiently immunogenic to protect from both severe and mild disease.

## 2. Materials and Methods

To identify eligible articles that describe attenuated coronaviruses, Scopus [73], Web of Science [74], Embase [75] and PubMed [76] databases were searched. The exact search keys used for each database are presented in Supplementary Materials S1. In short, they aimed to find articles that mentioned a coronavirus in the title alongside attenuation or a similar term in the title, abstract or keywords. This somewhat limited strategy was chosen because of the overwhelming volume of recent research regarding coronaviruses. Each database was last accessed on 12 February 2022. The results were merged, and duplicates were removed automatically by matching DOI numbers, PMID numbers + titles or publication years + titles + journal names. Preprints were removed based on journal names. Articles that had been found outside the search (through non-systematic reference-screening, search engine suggestions, search strategy testing) were also included. Each record identified in the search results was screened by title and abstract followed by full-text screening by two independent researchers. Varying assessments were settled by discussion or by a third researcher. The following eligibility criteria were used:

1. The article is written in English;
2. The article describes an actual and practical creation, acquisition or use of a coronavirus;
3. That coronavirus shows an attenuated phenotype in vivo such as decreased mortality, reduced symptoms, viral titer or load after infection relative to a virus from which it is derived. The infection may be either in a natural host of the virus or a suitable animal model;
  - Alternatively: That coronavirus lacks characteristics described in criterion no. 3, but its other properties stemming from some identified mutations are useful in the context of LAV;
4. The described coronavirus can be propagated with a measurable viral titer and no passage limit in permissive cell line or embryonated chicken eggs;
5. The nucleotide sequence of the coronavirus or amino acid sequence of its proteins are known and can be compared to the parental virus;
6. The attenuating mutations can be applied to SARS-CoV-2. That is, the differences between parental and attenuated viruses affect amino acid residues, whole proteins or nucleotide sequences present in SARS-CoV-2. In case of doubt, sequence alignment and literature data will be used to decide if the attenuation method is applicable;
7. The article presents data from in vivo studies.

The exclusion criteria were as follows:

1. The article describes the generation or acquisition of a coronavirus insufficiently, precluding replication of the results;
2. The article describes sequencing or genotyping of a coronavirus in infected humans or animals but not its isolation;
3. The described virus must be pretreated (with ionizing radiation, temperature, denaturing agents) before it can be used as a vaccine. That is, the specimen was only tested as a component of partially or fully inactivated vaccine;
4. The article is a review;
5. The article is a preprint;
6. The article presents only the data not useful in the context of this study, such as sequencing and structural data.

The focus of this study is to identify suitable animal studies, but human studies were not explicitly excluded should any been found. Coronavirus-derived replicons that can be packaged in coronavirus-like particles were also allowed. Sequence alignment described in the inclusion criterion no. 6. was performed using Unipro UGENE v. 41 (Unipro, Novosibirsk, NVS, Russia) [77] and the MAFFT tool [78]. The results were visually inspected and compared with literature data. The complete list of GenBank entries used to assess the applicability of mutations is presented in Table S1. Sequence alignments relevant to the included studies are presented in Figure S1a–S1af. Silent mutations and mutations in non-coding sequences were assumed to be inconsequential unless otherwise implied.

The data were extracted from relevant articles by one researcher. WebPlotDigitalizer 4.5 (Ankit Rohatgi, Pacifica, CA, USA) was used to extract data from plots [79]. Although coronaviruses, whose attenuation was assessed in vivo, were the focus of this study, data regarding attenuated coronaviruses that were only examined in vitro in the included studies were also extracted for the sake of completeness. The following data were extracted:

1. Mutations present relative to the WT virus;
2. Titers at the time the WT virus reached peak titer;
3. Results of in vitro studies;
4. Any determinants of pathogenicity of the viruses observed in vivo; mortality and weight loss reported as significant were extracted quantitatively;
5. Results pertaining to the immunogenicity of the attenuated viruses observed before challenge, quantitatively;
6. Any results of the studies aimed at studying the reversion to virulence;
7. Results of challenge studies; mortality and weight loss reported as significant were extracted quantitatively.

For the purposes of review, the data were imputed into separate columns within a spreadsheet. Data regarding the same coronavirus from different articles were grouped together. The most important outcomes are presented in Table 1, Table 2, Table 3 and Table S2a–e. Data such as age of the animals, doses of the virus and similar parameters were also extracted. There were no major restrictions on the types of outcomes presented, partially because this review was not aimed at quantitative analysis. However, serum 50% neutralizing titers were the preferred outcome related to humoral immunity. This systematic review was not preregistered, and no elaborate protocol was prepared and published because it did not aim to generate clinically relevant data. Some minor issues related to Preferred Reporting Items for Systematic Reviews and Meta-Analyses (PRISMA) guidelines [80] are briefly discussed in Appendix A, and the PRISMA 2020 checklist is presented in Table S3.

## 3. Results

**Figure 1:**
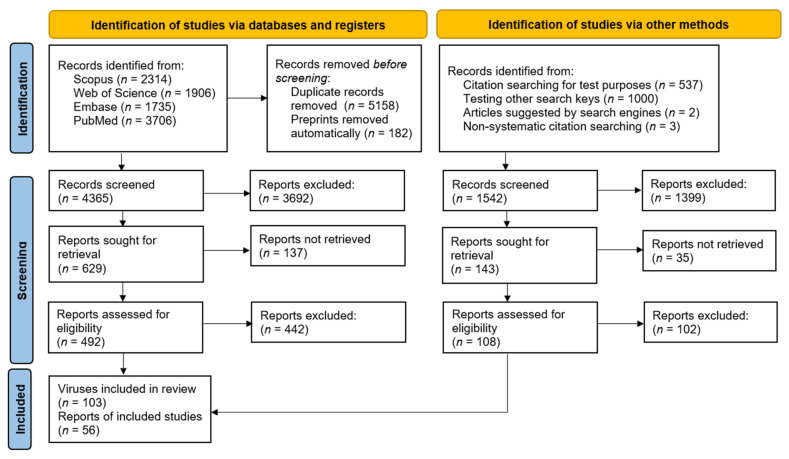
A simplified version of the Preferred Reporting Items for Systematic Reviews and Meta-Analyses (PRISMA) flowchart [80] of studies screened and included in the review.

A total of 2314 articles were retrieved from the Scopus database [73], 1906 articles were retrieved from the Web of Science database [74], 1735 articles were found in Embase [75] and 3706 were found in Pubmed [76]. In total, 56 papers were included, of which six papers were identified outside of the search. A detailed flowchart is presented in Figure S2, and a simplified version is presented in Figure 1. A complete list of excluded studies is also presented in Table S4.

There were a total of 101 attenuated coronaviruses described in the included papers, and 83 of them were tested in vivo. They were derived from seven viruses, namely: 29 viruses were derived from SARS-CoV, 28 viruses from SARS-CoV-2, 27 viruses derived MHV, six viruses from MERS-CoV, six viruses from IBV, three viruses from PEDV and two viruses from HCoV-229E, although none of the attenuated HCoV-229E viruses were tested in vivo. While several strains of SARS-CoV-2 were used as the starting point for attenuation, MHV-A59, MHV-JHM, SARS-CoV MA15 [81] and SARS-CoV Urbani were the genetic backgrounds of all attenuated SARS-CoV and MHV viruses.

**Figure 2:**
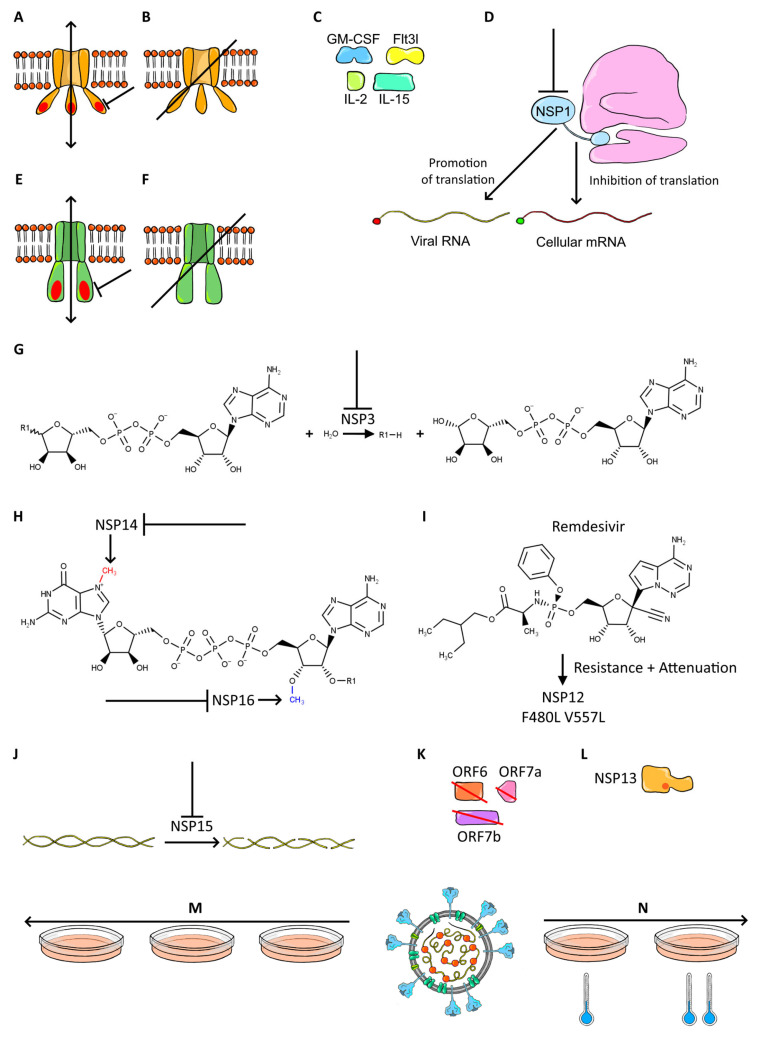
Attenuation strategies applicable to SARS-CoV-2 (Part 1). (**A**) Disruption of envelope ion channel and PDZ-binding domain. (**B**) Envelope protein deletion. (**C**) Coronavirus-encoded cytokines. (**D**) Disruption of NSP1 host-translation inhibitor. (**E**) Disruption of ORF3a ion channel and PDZ-binding domain. (**F**) ORF3a deletion. (**G**) Disruption of NSP3 macrodomain ADP-ribosylhydrolase activity. (**H**) Disruption of NSP14 N7-methyltransferase and NSP16 2′-O-methyltransferase. (**I**) NSP12 remdesivir-resistance mutations. (**J**) Disruption of NSP15 endonuclease. (**K**) Deletion of accessory proteins. (**L**) NSP13 A336V mutation. (**M**) Serial passage. (**N**) Cold adaptation.

**Figure 3:**
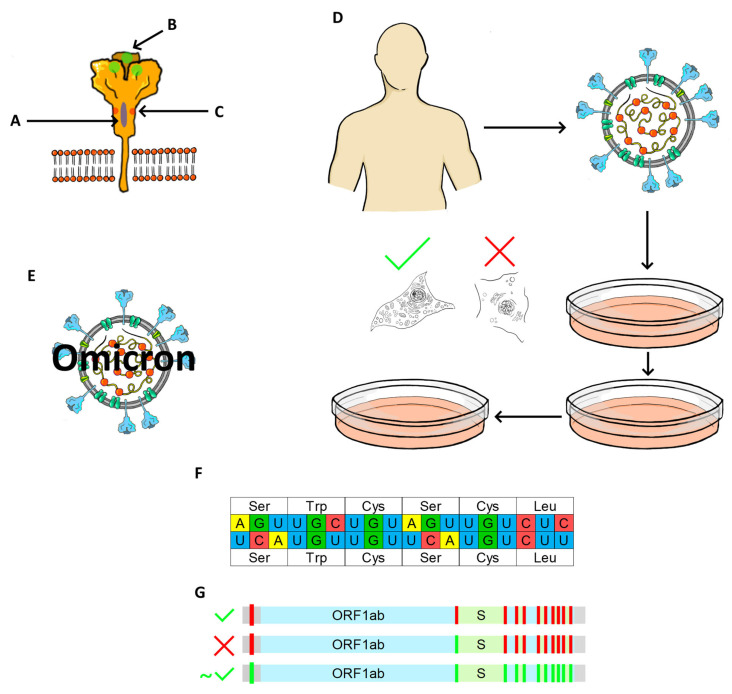
Attenuation strategies applicable to SARS-CoV-2 (Part 2). (**A**) Spike protein Heptad Repeat 1 disruption. (**B**) Spike T345I mutation. (**C**) Furin cleavage site disruption. (**D**) Naturally-occurring, persistently replicating SARS-CoV-2 isolate. (**E**) SARS-CoV-2 Omicron variant. (**F**) Codon pair deoptimization; a fragment derived from parental virus (top) and one of the attenuated viruses (bottom) are presented; the exchange of synonymous codons within a genome fragment is not shown. (**G**) TRS core replacements.

A total of 27 attenuation strategies, which can be or were applied to SARS-CoV-2, were identified. A full list is presented in Table 1, along with the degree of attenuation observed after the mutation was introduced. Attenuation strategies are also presented in Figure 2 and Figure 3. The attenuation degree was determined by change in infection mortality and symptoms. When there were no sufficient data on mortality or symptoms reported and only viral titers were presented, the attenuation degree was deemed unclear.

**Table 1:** A complete list of mutations known to attenuate coronaviruses, which can be applied to SARS-CoV-2.

Strategy	Relevant Mutations in SARS-CoV-2	Strategy is Known to Attenuate:	Degree of Attenuation	Ref.
Envelope PBM disruption	Envelope DLLV72-75GGGG	SARS-CoV MA15	Medium	[82,83,84]
Envelope IC disruption	Envelope N15A; Envelope V25F; Envelope Δ38–45; Envelope Δ46–52; Envelope: S3A, V5L, T9A, T11A;	SARS-CoV MA15	Low to high; V25F reverts easily	[82,84,85,86]
Envelope protein deletion	ΔEnvelope	SARS-CoV MA15; SARS-CoV Urbani; MERS-CoV; SARS-CoV-2	Medium to high	[83,84,85,86,87,88,89,90,91,92,93,94,95]
Coronavirus-encoded cytokine	Insertion of Flt3l, IL-2, IL-15 or GM-CSF genes	MHV-GP, MHV-MelA **	Unclear. Increased immunogenicity	[64,96]
NSP1 disruption	NSP1 K125R; NSP1: R124S, K125E; NSP1 Δ122–130	MHV JHM.WU; MHV-A59; SARS-CoV MA15	Medium or unclear	[84,97]
NSP3 macrodomain disruption	NSP3 D226A; NSP3 N244A; NSP3 H249A; NSP3 G334V; NSP3: A333G, G334V	MHV-JHM IA; MHV-A59; SARS-CoV MA15	Low to high; G334V reverts easily	[65,66,98,99,100,101]
NSP12 remdesivir-resistance mutations	NSP12: F480L, V557L	SARS-CoV MA15	Low	[49]
NSP13 A336V mutation	NSP13 A336V	MHV JHM.WU	Unclear	[97]
NSP14 N7-methyltransferase disruption	NSP14 D331A; NSP14 Y420A; NSP14 Y420H	MHV-A59; SARS-CoV-2	Medium to high; NSP14 Y420A: unclear	[102,103,104]
NSP14 V398L mutation	NSP14 V398L	IBV-M41-derived virus	Low	[105]
NSP15 endonuclease disruption	NSP15 H234A; NSP15 H249A; NSP15 K289A; NSP15 Y342A	PEDV; IBV, MHV-59	Medium to high	[106,107,108,109]
NSP16 2′-O-methyltransferase disruption	NSP16 Y15A; NSP16 K46A; NSP16 D130A; NSP16 K170A; NSP16 E203A	PEDV; MHV-A59; SARS-CoV MA15; SARS-CoV Urbani; MERS-CoV	Low to high SARS-CoV MA15 in aged BALB/c mice at 10^5^ PFU: no attenuation	[34,70,71,72,110]
ORF3a PBM disruption	ORF3a SVPL272-275GMSM	SARS-CoV MA15	Low	[56]
ORF3a ion channel disruption	ORF3a: S40A, S58A; ORF3a: Y109A, Y113A, Q116A; ORF3a: Y91A, H93A	SARS-CoV MA15	Low to medium	[56]
ORF3a deletion	ΔORF3a	SARS-CoV MA15; SARS-CoV-2	Low to medium	[82,95,111]
ORF6 deletion	ΔORF6	SARS-CoV-2	Low	[111]
ORF7a deletion	ΔORF7a	SARS-CoV-2	Low	[111]
ORF7b deletion	ΔORF7b	SARS-CoV-2	Low	[111]
Spike T345I mutation	Spike T345I	SARS-CoV Urbani	Low	[48]
FCS disruption *	See Figure 4	SARS-CoV-2	Medium to high	[5,36,112,113,114,115,116]
HR1 mutation	Spike: L1012R ± Q965H and Q992H,	MHV-A59 with JHM spike	Unclear	[117,118]
TRS core replacement	ACGAAC to: CCGGAU or UGGUCGC	SARS-CoV Urbani, SARS-CoV MA15	Low to high	[95,119]
Codon pair deoptimization	Exchange of synonymous codon positions	SARS-CoV-2	None to high	[5,120,121]
Serial passage and cold adaptation	See Table S2a	SARS-CoV-2	High	[122]
Serial passage *	See Table S2a	SARS-CoV-2	Medium to high	[36]
Naturally occurring persistently replicating isolate	See Table S2a	SARS-CoV-2	Unclear	[123]
Omicron variant	See Table S2a	SARS-CoV-2	Low to high	[124,125,126]

The following definitions were used to assess the degree of attenuation: high: fails to cause death, weight loss or other symptoms; medium: fails to cause death, but animals experienced only reduced weight loss or other symptoms; low: causes death, although less consistently than WT virus or minimal reduction in weight loss or other symptoms; unclear: the infected animals were not observed for long enough to observe death, or no sufficient data regarding weight loss or symptoms were available and the virus was determined to be attenuated due to reduced titer or a similar parameter. If the lethality or symptomatic disease were caused by reversion, the degree of attenuation was judged in the unmutated virus, but this was noted. * Passaging SARS-CoV-2 in Vero cells usually results in the loss of FCS. ** MHV-GP and MHV-MelA are highly attenuated MHV-A59-based vectors that express green-fluorescent fusion protein with the lymphocytic choriomeningitis virus gp33-41 epitope or the Mel-A26-35 epitope, respectively. NSP, non-structural protein; ORF, open reading frame; PBM, PDZ-binding-motif; FCS, furin cleavage site; TRS, transcriptional regulatory sequence; HR1, heptad repeat 1.

The viruses were evaluated in several animal models; the most important ones are listed in Table 2. SARS-CoV-2 infection was usually assessed in K18-hACE2 mice, for which infection with the WT virus is lethal [127], and Syrian hamsters, for which infection is symptomatic [128]. The most common model for SARS-CoV infection was a combination of BALB/c mice and SARS-CoV MA15; the infection is lethal at higher virus doses [81]. Because SARS-CoV-2 and SARS-CoV are related, SARS-CoV MA15 may serve as the most useful approximation for mutations not examined in SARS-CoV-2. IBV and PEDV were tested in their natural hosts—chickens and piglets—while MHV was administered to various strains of mice, usually C57BL/6. The routes of MHV administration varied and included intranasal, intraperitoneal, intrahepatic, intravenous, intracranial, intramuscular and subcutaneous. The behavior of coronaviruses with corresponding mutations differed in animal models; while most MHV mutants did not appear to cause significant pathology [65,70,102,103,104,107], PEDV was able to cause disease of only reduced severity despite attenuating mutations [72,97].

**Figure 4:**
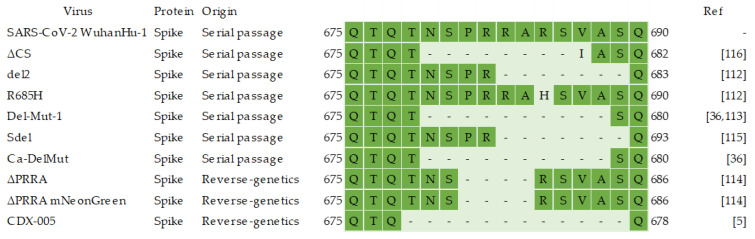
A comparison of the FCS deletions and substitutions in the attenuated SARS-CoV-2 viruses [5,36,112,113,114,115,116].

The most promising strategy for SARS-CoV-2 attenuation was the deletion of the FCS. FCS is consistently lost when the virus is passaged in Vero E6 cells, but it can also be deleted using reverse genetics [5,36,112,113,114,115,116,117,118,119,120,121,122,123,124,125,126,129,130,131,132]. A sequence alignment of the FCS regions of all FCS mutants is presented in Figure 4. SARS-CoV-2 viruses, which lack FCS, were as immunogenic as the WT virus in ferrets and Syrian hamsters but were not transmissible [36,112,114,115,116]. In Syrian hamsters, they caused no symptoms and minimal lung inflammation but protected them from infection with lineage P.1 and lineage B.1.1.7 viruses [36,112,113,114,115]. One such virus caused K18-hACE2 mice to lose some weight and experience mild changes in pulmonary mechanics [114]. This indicates that a strategy combining FCS deletion and some other attenuating mutations is preferable to prevent disease in more susceptible humans; for example, FCS deletion and codon deoptimization may be combined [5].

**Table 2:** The models of coronavirus infection identified in the included studies, excluding animals with innate and adaptive immune deficiencies.

Virus	Animal	Route of Inoculation	Disease Course *	Reference
IBV	Chickens	Conjunctival ± intranasal	Symptomatic or lethal	[105,106]
MHV-A59, MHV-JHM	C57BL/6 mice, 129Sv mice	Intranasal, intraperitoneal, intrahepatic, intravenous, intracranial, intramuscular or subcutaneous	Lethal (except for subcutaneous administration)	[64,65,66,70,102,103,104,107]
PEDV	Piglet	Oral	Lethal	[72,108]
SARS-CoV MA15	BALB/c mice	Intranasal	Lethal	[34,82,83,84,85,86,88,89,93,98,101,119]
SARS-CoV Urbani	Syrian hamster	Intranasal	Symptomatic	[90,91]
	hACE2-Tg mice	Intranasal	Lethal	[92,96]
	BALB/c mice	Intranasal	Symptomatic	[48,88]
SARS-CoV-2	Ferret	Intranasal	Asymptomatic	[116]
	Syrian hamster	Intranasal	Symptomatic	[5,36,95,112,113,114,115,120,124]
	Roborovski dwarf hamster	Intranasal	Symptomatic	[120,121]
	K18-hACE2 mice	Intranasal, intracranial	Lethal	[95,103,111,114,122,124,125]
	K18-hACE2 Syrian hamster	Intranasal	Lethal	[124]
	Ad5-hACE2 transduced BALB/c mice	Intranasal	Not reported in the included studies **	[36]
SARS-CoV-2 variants of concern	129S1, BALB/c and C57BL/6 mice	Intranasal	Lethal	[124,126]
MERS-CoV EMC	Ad5-hDPP4 transduced BALB/c mice; Dpp4 288–330^+/+^ mice	Intranasal	Asymptomatic	[71]
	K18-hDPP4 mice	Intranasal	Lethal	[87]
MERS-MA30	hDPP4-KI mice	Intranasal	Lethal	[87]
MERS-CoV MA1	Dpp4 288–330^+/+^ mice	Intranasal	Lethal	[71]

* The most common disease course in the included studies. Disease course may be obstructed by the fact that animals experiencing severe disease were sometimes euthanized. This was particularly visible in the case of the Roborovski dwarf hamster model, which experienced a very severe disease but were always sacrificed before their natural death in all published reports. ** Studies that did not involve SARS-CoV-2 attenuation reported symptomatic disease.

The reverse genetics techniques were used to introduce several attenuating mutations into coronaviral genomes. They mostly centered around abrogating innate immunity evasion and disabled such functions as the NSP1 host protein translation inhibitor [85,97], NSP3 macrodomain activity [65,66,98,99,100,101], NSP15 endoribonuclease [106,107,108,109] and NSP16 2′-O-methyltransferase activity [34,70,71,72,110]. They also involved deletions of the envelope protein (E protein) [83,84,85,86,87,88,89,90,91,92,93,94,95] or accessory proteins [82,112]. All of these mutations varied in the degree of attenuation and capacity of the attenuated viruses to revert to virulence. A summary of the experiments that evaluated the capacity of attenuated coronaviruses to revert to the virulent phenotype in vivo is presented in Table 3. In the Table S2c, a summary of in vitro experiments in which the stability and capacity of the virus to revert to virulence were reported is presented. Although some of the viruses were stable after a single or even multiple passages, reversion after even more subsequent passages may still be possible.

Interestingly, two types of attenuating mutations that did not result in altered amino acid sequences were identified. First, the synonymous codons within a defined region of the viral genome may be exchanged. This leads to suboptimal codon pairs in a process known as codon pair deoptimization. The resulting virus exhibits less efficient protein synthesis and reduced viral fitness and pathogenicity [5,119,121]. Second, all transcriptional regulatory sequences (TRS), which are responsible for the synthesis of subgenomic RNA strands, share a common core sequence within a single coronaviral genome. These core sequences may all be replaced [95,119], which attenuates the virus and also prevents the recombination with WT viruses, because a functional coronaviral genome requires all TRS core sequences to be identical [133].

**Table 3:** A summary of the results of experiments that aimed to study the reversion to virulence of attenuated coronaviruses in vivo.

Virus	Mutations *	Conditions	Results	Reference
SARS-CoV Urbani	TRS replacement (ACGAAC to CCGGAU)	6 passages in 14-month-old female BALB/c mice (5 experiments)	Virus caused lethal disease in some or all mice at passage 4, 5 and 6 in all 5 experiments. Vast deletions in Orfs 7b, 8a and 8b	[119]
SARS-CoV MA15	TRS replacement (ACGAAC to UGGUCGC)	4 passages in 10-week-old female BALB/c mice	No difference in weight loss or lung viral titer	[119]
SARS-CoV MA15	TRS replacement (ACGAAC to UGGUCGC)	6 passages in aged female BALB/c mice	No increase in mortality of infection in 12-month-old BALB/c mice	[119]
SARS-CoV MA15	NSP16 D130A	30 days of infection in female RAG^−/−^ mice	Reversion in 5/8 mice, probably due to synonymous mutations	[110]
SARS-CoV MA15	ΔEnvelope	10 passages in 16-week-old female BALB/c mice	Orf8a gene was partially duplicated, and the resultant protein contained a PDZ-binding motif. Infection with this virus was lethal.	[91]
SARS-CoV-2	Spike R685H	5–6-week-old Syrian hamster infection	No reversion	[112]
SARS-CoV-2	Spike Δ683–689	5–6-week-old Syrian hamster infection	No reversion	[112]
SARS-CoV MA15	Envelope N15A	16-week-old female BALB/c mice	No reversion	[70]
SARS-CoV MA15	Envelope V25F	16-week-old female BALB/c mice	Several reverting mutations appeared at 2 days post infection	[70]
IBV M41R-nsp10.14rep	NSP14 V398L	8-day-old chickens	No reversion	[114]
SARS-CoV MA15	ORF3a: S40A, S58A	16-week-old female BALB/c mice	No reversion in deceased mice	[82]
MHV-JHM IA	NSP3 D226A	5–8-week-old C57BL/6 mice	Revertant viruses found in brains of deceased mice: D497A or L481V + K495E mutations found	[66]
MHV-A59	NSP14 Y420H	4-week-old C57BL/6 mice	Virus reverted in one out of four infected mice	[129]

* Mutations listed are corresponding mutations in SARS-CoV-2. Actual mutations are presented in the Table S2a and Figure S1a,af.

Based on the results of the animal studies, the attenuated SARS-CoV-2 virus would be a highly effective COVID-19 vaccine. The intranasal route of administration appears to be the most suitable, as it was utilized in most of included studies involving SARS-CoV-2. Sufficiently attenuated SARS-CoV MA15 and SARS-CoV-2 viruses caused only minor inflammation in vaccinated animals (Table S2b). The immunogenicity was usually high; a summary of the results related to immunogenicity is presented in Table S2d. Highly sensitive BALB/c and K18-hACE2 mice were universally protected against lethal disease and significant weight loss after immunization with several SARS-CoV MA15 mutants or one of the SARS-CoV-2 mutants followed by challenges with the WT virus [103]. A summary of challenge studies is presented in Table S2e. Therefore, it may be concluded that inoculation with sufficiently attenuated SARS-CoV-2 would likely cause asymptomatic pneumonia in humans at most, while the protection against symptomatic disease would be high.

## 4. Discussion

The role of LAV in the context of the COVID-19 pandemic primarily needs to be to prevent transmission to the most susceptible individuals, who are not eligible for LAVs themselves. Other benefits may include the reduced impact of isolation of the diseased patients on the economy and reduction in rarely occurring severe cases of COVID-19 in individuals without significant risk factors. It must also be remembered that LAV would need to be rapidly developed in the pandemic setting.

There are generally two methods of obtaining attenuated coronaviruses: reverse genetics and serial passage. Regarding the former, it was reported that a SARS-CoV-2 clone could be obtained through reverse genetics within a month or less [61]. When it comes to attenuation through serial passage, cell-culture adapted and cold-adapted SARS-CoV-2 can be obtained in roughly 30 to 60 days [36,113,121], while an attenuating FCS disruption occurs very rapidly in Vero cell culture [5,36,112,113,114,115,116,129,130,131,132]. In general, attenuated SARS-CoV-2 can be obtained or engineered reasonably quickly in the pandemic setting, unless a very heavily passaged virus is desired.

### 4.1. Models of Coronavirus Infection

While, based on some published research, it is evident that attenuated SARS-CoV-2 virus can be rapidly created, its potential safety profile requires a more careful examination. It must be remembered that COVID-19 is non-fatal for the majority of individuals [134]. Therefore, it is very likely that the attenuation of SARS-CoV-2 in an animal model of lethal infection would be sufficient to demonstrate adequate attenuation for practical use in healthy individuals at low risk of severe COVID-19. An animal model of lethal disease is also necessary to determine the outcome of inadvertent administration of the vaccine to a susceptible individual, which at least should not be lethal. K18-hACE2 mice are a commonly used model of lethal COVID-19 [127], although they sometimes display a more pronounced neuroinfection [135,136,137,138] and are particularly sensitive to intracranial inoculation [95]. Roborovski dwarf hamsters are reported to experience a very human-like infection, but it is unclear if they succumb to the disease if not euthanized [139]. Other less susceptible animals, such as Syrian hamsters and hACE2-Tg mice, may serve as models for a more typical disease course in the target population [128,140]. Non-human primates (NHPs), such as rhesus macaques or cynomolgus macaques, were not used in the reviewed studies but would have provided the most human-like model of attenuated SARS-CoV-2 infection [141,142,143]. Ferrets are not very susceptible to SARS-CoV-2 infection, although some conflicting reports exist [144,145,146] and are probably most useful in studying the transmission of the vaccine virus [146,147].

Based on the information presented in the previous paragraph, animal models might be used to predict the properties of an attenuated COVID-19 vaccine. However, a large subset of studies regarding attenuated coronaviruses did not concern SARS-CoV-2. If the data collected using animal infection models with different coronaviruses are to be applied to SARS-CoV-2, it is necessary to understand some crucial characteristics of those models. Some of the mutations in the included viruses, other than those derived from SARS-CoV-2, affected highly conserved functions regulating the evasion of innate immunity. These consistently yielded attenuated phenotypes in different viruses. More caution is perhaps to be required if the attenuation stems from altered tropism. MHV-A59 expressing an MHV-JHM spike showed markedly reduced neurovirulence after the spike L1114R mutation was introduced, especially in combination with Q1067H and Q1094H [117,118]. Furthermore, in a study by Zhang et al. [97], two MHV-JHM-related strains, MHV-JHM.WU and MHV-JHM.SD, were compared; MHV-JHM.SD failed to replicate efficiently in liver but achieved only slightly reduced brain viral titer in mice when compared to MHV-JHM.WU. When some polymorphisms (NSP1 K194R and NSP13 A335V) present in MHV-JHM.SD were introduced into an MHV-JHM.WU-like virus, the viral replication in the liver was diminished, but there is no data on the brain viral titers or overall lethality and weight loss that mice experienced [97]. Such results are difficult to extrapolate to SARS-CoV-2, which seems not to be as neurovirulent [148] and does not readily replicate in the liver in most individuals [149]. In fact, SARS-CoV-2, SARS-CoV MA15 and Urbani and MERS-CoV are primarily respiratory viruses, but MHV-A59 and MHV-JHM have a broader tropism and replicate effectively in the brain, spleen and liver [149]. The scale of attenuation used in this work reflects this issues. It was assumed that a mere reduction in titer in one of the organs may not translate to any clinically relevant outcome and, therefore, the degree of attenuation is defined based on the reduction in mortality and symptoms of the infection.

Despite the broad tropism of MHV, most attenuated viruses derived from it were not very pathogenic. On the other hand, PEDV, an enteric virus, appeared to be less sensitive to attenuating mutations than the aforementioned viruses [72,108]. It was not possible to determine if the IBV virus had a similar tendency to stay virulent after an attenuating mutation was introduced due to a lack of sufficient data. In summary, data regarding PEDV, MHV and IBV should serve a secondary role in vaccine design against SARS-CoV-2.

While the included studies described experiments in young and aged animal models, there were no relevant immunosuppression models except for TNF^−/−^ mice. Interestingly, they most often died but did not lose weight upon MHV-A59 infection. Both weight loss and mortality were not noted when the virus had the NSP3 N515A mutation. This may suggest that the attenuated SARS-CoV-2 vaccine would be safe for patients receiving TNF inhibitors [100]. As far as defects in innate immunity are concerned, only rather obscure models such as IFNAR1-deficient mice and TLR7-deficient mice were studied. Polymorphisms in those genes were associated with severe or unusual COVID-19 in humans, but it is unclear whether those models are truly relevant [70,96,150,151]. Inborn errors of acquired immunity are also associated with severe COVID-19 [152]. Some RAG1^−/−^ mice were able to clear the NSP3 N515A MHV-JHM IA mutant and NSP16 D130A SARS-CoV MA15 mutant, but others died or developed chronic infection with the revertant virus [100,110]. This indicates that the attenuated SARS-CoV-2 vaccine virus will likely be unsafe for individuals with severe immune deficiencies.

### 4.2. Attenuation Strategies

As the models of coronavirus infections were discussed in the previous paragraphs, the following part will focus on the targets of attenuation methods.

Coronaviruses encode a few conserved proteins involved in preventing the detection of viral pathogen-associated molecular patterns and the disruption of interferon signaling or the antiviral functions of interferon-stimulated genes. Most of the attenuation strategies in this review involved mutations that disabled those functions. Betacoronavirus NSP1 is a protein primarily involved in inhibiting the translation of and degrading host mRNA [153]. This also leads to the inhibition of translation of antiviral signaling proteins such as Tyk2 and STAT2 [154]. The macrodomain of NSP3 protein is known to have ADP-ribosylhydrolase activity and to remove mono-ADP-ribose (MAR) and poly(ADP-ribose) (PAR) chains from proteins in vitro [155]. It was shown to counteract the activity of poly(ADP-ribose) polymerases (PARPs), which are known to inhibit viral infections. In the case of MHV-JHM, PARP12 and PARP14 were shown to be particularly important [99]. For SARS-CoV-2, the macrodomain was shown to counteract the activity of PARP9 [156]. Therefore, the exact functions of the NSP3 macrodomain may differ between individual cells and coronaviruses and are not clearly elucidated.

Coronaviral NSP14 has two enzymatic activities. Its proof-reading exonuclease activity is not dispensable in SARS-CoV-2 [157]. However, full N7-methyltransferase activity is necessary only for virulence. It is involved in capping viral RNA, and it is not currently clear if viable attenuated viruses with NSP14 defects have some residual N7-methyltransferase activity. The exact roles of NSP14 mutations in coronavirus attenuation is further obscured by the fact that they yield different phenotypes in different coronaviruses [103,158].

NSP15 and NSP16 are yet other proteins involved in coronaviral immune evasion. NSP15 has endonuclease activity, which prevents dsRNA sensing [106,159,160], while NSP16 is responsible for 2-O’-ribose methylation in viral cap synthesis. NSP15 seems required to evade sensing by OAS1-3, PKR and MDA5 [106,109]. Meanwhile, 2-O’-ribose methylation was necessary for evading recognition by MDA5, TLR7, IFIT1 and IFIT2 [34,70]. The 2-O’-ribose methylase-deficient SARS-CoV MA15 displayed an interesting phenotype, where immune response profile is similar to that of the WT virus in the initial phase of the infection in vivo and in vitro. Unsurprisingly, infection with this virus was lethal to aged BALB/c mice at higher doses, which indicates that the disruption of NSP16 activity alone is not sufficient for vaccination purposes [110].

The next target of attenuating mutations was the envelope protein, which was intensively studied in SARS-CoV. Disruptions in the E protein ion channel and PDZ-binding motif (PBM) were attenuating, as was the deletion of the whole envelope protein. The exact role of the ion channel activity of the SARS-CoV E protein is not very well understood, but its involvement in inflammasome activation was well-proven and may be a mechanism involved in the virulence of the virus [86,161]. Several functions were postulated for PBM, but one of the most compelling and proven is shifting the localization of syntenin to the cytoplasm, which activates p38 MAPK and triggers inflammatory cytokine expression [83,161]. The envelope protein of SARS-CoV is also clearly involved in virion assembly, budding and structure. SARS-CoV MA15, which lacked the E protein, produced a reduced number of viral particles in the cytoplasm of Vero cells. Those particles were more often captured during budding and disrupted or aggregated in the supernatant concentrate [91,94]. Overall, E protein is an attractive target for SARS-CoV-2 attenuation. Out of all mutations described in the reviewed studies, envelope Δ46–52 may be considered the most attractive target since it was rather highly attenuating. It did not revert as quickly as E protein deletion, point mutations disrupting the ion channel or the deletion of PBM [86].

Regarding the FCS mutants, it was established that they are less fit in TMPRSS2-expressing cells [114,115,116]. It was also shown that those mutants enter cells not through the membrane as the WT virus does but through fusion in endosomes [115,116]. On the one hand, this mechanism is dominant in Vero E6 cells, and the FCS mutants may be more fit in them due to the enhanced stability of the spike protein lacking FCS [114,116]. On the other hand, the endosomal entry pathway exposes the virus to IFITM proteins that restrict membrane fusion [116,162]. Interestingly, at least one FCS mutant showed an increased tendency to form clusters of viral particles. The authors hypothesized that clustering was the reason for decreased neutralization of that virus by human sera and monoclonal antibodies [114]. The impact of this finding on the purification and processing of a vaccine virus is not clear.

Based on the arguments presented in Section 3, FCS deletion appears to be the best strategy for achieving an attenuated but replicating SARS-CoV-2 vaccine. This finding is accompanied by two interesting conclusions. First, FCS is present in SARS-CoV-2 spikes but not in spikes of closely related coronaviruses, such as SARS-CoV [163]. Therefore, universal attenuation strategies broadly applicable to many coronaviruses may not always be the most optimal option. Second, any other attenuation strategy would need to account for FCS deletions or disruptions that arise spontaneously in Vero cell culture [129,130,131,132]. This could pose a problem for the production of a non-FCS-deleted vaccine virus in typical Vero cells, such as the widely recognized WHO Vero RCB 10–87 cell line, which is available free-of-charge to vaccine manufacturers from WHO [164]. It would need to be overcome by using TMPRSS2-expressing Vero cells or other cell lines to maintain genetic stability and consistency of vaccine viruses with preserved FCS [132].

The final protein targets that were altered in attenuated mutants are accessory proteins. Their deletions are only weakly attenuating in SARS-CoV-2 [111]. They differently altered cytokine response in K18-hACE2 mice, and open reading frame 3a (ORF3a) deletion resulted in the lowest IL-6/IL-10 ratio, a marker of cytokine storm in COVID-19. Unsurprisingly, the lack of ORF3a also leads to the least virulent virus [111]. While weakly attenuating on their own, deletions of accessory proteins may be useful if combined with other attenuating mutations, but they could also serve another purpose. It appears that coronaviral accessory proteins are involved in the reversion to virulence, as was the case with the SARS-CoV TRS mutant [119] and envelope-deleted SARS-CoV [84]. It was also found that SARS-CoV-2 isolates from infected minks had mutations in ORF3 and ORF7a deletion [165]. Perhaps if accessory proteins were deleted, some capacity of SARS-CoV-2 to adapt to new hosts and revert to virulence could be lost.

The strategies mentioned above alone did not entirely prevent the propagation of the viruses in vaccinated animals. However, the development of an attenuated or attenuated-like COVID-19 vaccine for immunocompromised patients that mimics the properties of highly attenuated MVA appears feasible. Simultaneous deletion of ORF3a and E proteins in SARS-CoV-2 leads to packaging-deficient replicons that require complementation with E and ORF3a proteins in trans to produce infectious progeny and cannot propagate in normal mammalian cells [82,95]. They did not cause disease even after intracranial inoculation in K18-hACE2 mice. However, a minimal tendency to adapt to the lack of E and ORF3a was present. After five consecutive passages in one of the experiments, an avirulent virus, which could replicate to a low titer in Vero E6 cells, was obtained [90]. Various packaging-deficient MERS-CoV-derived replicons lacking E protein were also obtained, and they too could produce infectious particles when E protein was supplied in trans. They appeared to grant susceptible mice a high degree of immunity from lethal MERS-CoV infection, but one of them, derived from the strain MERS-CoV EMC/2012, appeared to cause some weight loss in K18-hDPP4 mice [87].

Interestingly, based on animal studies, it has been shown that the SARS-CoV-2 variant of concern Omicron, lineage BA.1 (also known as B.1.1.529.1, previously referred to as B.1.1.529 [166,167]) is attenuated. Nevertheless, its pathogenicity was still unacceptably high for vaccination purposes [124,125,126]. Additionally, increased transmissibility in humans could be an issue for LAVs derived from Omicron [168], even if additional mutations were included to attenuate the virus further.

As it has been demonstrated, several attenuating mutations can be introduced into the SARS-CoV-2 genome through the use of reverse genetics. However, safe use of the vaccine will not depend solely on the lack of pathogenicity of the vaccine virus. New variants may arise if the WT and vaccine SARS-CoV-2 are allowed to recombine in human host cells, as it was in the cases of other coronaviruses. In fact, recombination between the vaccine and WT strains of IBV seems to increase the diversity of circulating strains [38,39,40,41]. There are several steps that can be undertaken to reduce the likelihood of a recombinant virus appearing from WT-vaccine recombination. One already mentioned strategy is TRS replacement [119]. If two attenuating mutations are placed in distant parts of the coronavirus genome, most recombinant coronaviruses would inherit at least one and not be fully virulent and infectious [72]. As recombination in coronaviruses is driven by homology, it would likely mean that larger deletions may slightly decrease the likelihood of recombination events. For example, ORF3a is encoded by 828 nucleotides, and when those are deleted, the recombination may not happen in that region. Codon pair deoptimization may also prevent recombination if the homology between WT and the deoptimized sequence is sufficiently low. In a study by De Haan et al. [169], MHV viruses with a reshuffled S-E-M-N gene order were created. Such viruses would also be recombination-resistant, as a single recombination event would lead to a virus with deletions or duplications of those genes.

In reference to the existing data from human studies, there was one SARS-CoV-2 virus for which such data were available. An interesting isolate that displayed a minimal cytopathic effect was found in an asymptomatic patient who presented a high viral load. Vero E6 cells infected with this virus were viable and could be passaged. Such a persistently replicating variant would allow for obtaining more vaccine doses from a single batch of cells [123].

As mentioned before, combining two or more attenuation strategies may be desirable to increase the degree of attenuation. This was performed for only five of the included viruses; FCS deletion was combined with codon deoptimization while PBM was deleted from ORF3a and E protein simultaneously [5,82]. No clear conclusions can be derived regarding those combinations, as that particular instance of codon deoptimization was not tested alone, and ORF3a PBM had little effect on virulence on its own [5,82]. Three SARS-CoV-2-derived replicons had simultaneous E and ORF3a deletions, along with TRS replacement, but this, as was discussed before, leads not merely to attenuation but to the inability to produce infectious particles [95]. Nevertheless, some strategies that cannot be directly applied to SARS-CoV-2 were combined in PEDV and SARS-CoV [34,72,84,111]. In those cases, the viruses with combinations of mutations were more attenuated and less likely to revert than viruses with a single mutation.

### 4.3. Potential Profile of Attenuated COVID-19 Vaccine

The next issue discussed herein will be the probable safety and efficacy profile of the attenuated SARS-CoV-2 vaccine. Attenuated SARS-CoV and SARS-CoV-2 usually caused mild lung inflammation and sometimes minimal lung damage in animals, as evidenced by histopathological examinations. While certainly not desirable, it does not preclude the applicability of the attenuated SARS-CoV-2 vaccine. The attenuated influenza vaccine was also noted to cause pulmonary inflammation in some ferrets in preclinical studies [170]. Any problems related to pneumonia induced by the vaccine virus could probably be avoided by simply ensuring sufficiently strong attenuation and potentially by choosing cold adaptation as an attenuation strategy that would restrict replication in lungs. Nevertheless, cold-adapted SARS-CoV-2 still caused mild pneumonia in K18-hACE2 mice at a higher dose [122].

Considering the immunogenicity of attenuated coronaviruses, it was usually high and similar to the WT virus (Table S2d). A notable exception was SARS-CoV Urbani with an E protein deletion. This virus was poorly immunogenic in BALB/c mice, unlike SARS-CoV MA15 with the same mutation. The presumed reason was a poor adaptation of SARS-CoV Urbani to replication in mice [88]. The same virus, along with the variant devoid of ORF6-9b, was also poorly immunogenic in hACE2-Tg mice because serum neutralizing titers were very low, and it offered limited protection in those animals. This is more surprising, as hACE2-Tg mice are very susceptible to SARS-CoV Urbani infection, which means that a robust replication and high immunogenicity of the attenuated viruses may be expected. However, the dose used in the challenge study was 50 times greater than LD_50_ [92].

An interesting finding related to immunogenicity is that SARS-CoV Urbani and SARS-CoV MA15 with the E protein deletion and SARS-CoV Urbani with TRS replacement were more immunogenic in aged animals [88,119]. This finding contrasts with the reduced immunogenicity of most vaccines in aged individuals [171]. It may result from an increased replication of the attenuated virus in aged animals with a less robust initial immune response.

Regarding the efficacy of the attenuated COVID-19 vaccine, the most convincing reports come from challenge studies (Table S2e). SARS-CoV MA15 and SARS-CoV-2 viruses protected animals from weight loss and mortality after a lethal challenge with high doses of the virus. Therefore, it may be inferred that attenuated SARS-CoV-2 would similarly protect human patients from symptomatic COVID-19 disease. There are fewer data available regarding the ability to prevent transmission and asymptomatic infections, but viral titers in the lungs and nasal turbinates of vaccinated animals were often not detectable after the challenge. There were also a few cases where only reduced viral titers were measured after the administration of the virulent virus at two days post-infection [5,36,88,114,121]. In the challenge studies, almost all attenuated coronaviruses granted significant protection, barring the aforementioned SARS-CoV Urbani with the E protein deletion. On the other hand, no clear correlation between the degree of attenuation and protection was observed.

Nevertheless, challenge studies have a peculiar feature related to infectious doses [71,72,119]. While a high dose of the virus, such as 10^6^ plaque-forming units (PFU), may be easily used to vaccinate individuals, it is unclear if such an amount of SARS-CoV-2 or other coronaviruses can actually be encountered during real-life exposure. Meanwhile, in the challenge studies, the vaccine and challenge doses may be similar. One reason for this is to increase the severity of the disease and mortality in the particular animal model. An already mentioned study did not find SARS-CoV Urbani with the envelope deletion to be protective when the challenge dose was 50 times higher than LD_50_ [92]. Nonetheless, it is difficult to extrapolate studies using different infectious doses from animals to humans. The exact infectious dose of SARS-CoV-2 in humans is not fully established, but in a human challenge study, 10 TCID_50_ (roughly equivalent to 55 FFU) was sufficient to infect 53% healthy, SARS-CoV-2-naïve individuals [172]. The exact dose typically encountered during exposure was once estimated to be in the range of hundreds of PFUs [173]. The 6–8-weeks-old K18-hACE2 mice (with C57BL/6J background) appeared to be more sensitive to infection, because 20 PFU was enough to cause 80% mortality [135].

An interesting aspect of the attenuated COVID-19 vaccine may be the choice of the route of administration. Intranasal appears to be the most obvious. It mimics the natural mode of infection and delivers the virus to the tissues where it can efficiently replicate. In fact, the human challenge study of virulent SARS-CoV-2 used nasal drops to deliver the challenge virus [172]. It is also the only route studied for attenuated SARS-CoV-2 viruses, aside from the impractically difficult and dangerous intracranial route [95]. Unfortunately, involvement of the lower respiratory tract may lead to adverse outcomes, particularly if attenuation is weak. Intradermal and intramuscular administration may also be feasible. SARS-CoV-2 was shown to replicate in sweat glands and dermal endothelia [174], while SARS-CoV-2 RNA, including antisense RNA indicative of active replication, was found to be present in skeletal muscles of deceased persons with COVID-19 [6,175,176]. The intradermal route may have an additional advantage of enhancing vaccine immunogenicity [177]. On the one hand, intradermal or intramuscular administration may offer some safety-related benefits because they could prevent the involvement of critical organs, such as lungs, in the infection with the vaccine virus [178,179]. On the other hand, the elicitation of mucosal immunity by this route may be limited. Whether dermis or muscles can support sufficient virus replication for the elicitation of strong immunity remains uncertain. Infectious particles could be immunogenic without replication akin to inactivated SARS-CoV-2 virions found in some marketed vaccines. Even so, administering live viral particles that will not replicate may defeat the point of LAV. Rhesus macaques could not be infected via gastric route, which likely precludes vaccination with oral drops, but limited infection was observed after post-pyloric administration [180,181]. The immunogenicity of WT SARS-CoV-2 administered this way was low and did not provide full protection. This limits the potential of enteric capsules or tablets as a method of administering SARS-CoV-2 LAV [181]. Infection via the conjunctival route is possible in rhesus macaques [180]. Needless to say, higher purity standards than for the intranasal route would be required, and the risk of ocular complications likely precludes the administration of SARS-CoV-2 LAV as eye drops.

Comparing the potential profile of attenuated SARS-CoV-2 and currently used COVID-19 vaccines leads to an important issue that has not been answered by using animal models so far. Almost all currently used COVID-19 vaccines require more than one dose for full efficacy and booster doses due to waning immunity [182]. Meanwhile, the included studies did not involve experiments aimed at testing the second or booster doses of vaccine viruses. Immunity gained after the first dose will likely limit the replication and, therefore, the immunogenicity of the attenuated virus administered as a second or booster dose. Perhaps this may not be a significant issue, as some degree of replication of virulent viruses was still observed in a few challenge studies [5,36,88,114,121]. Another approach may be to use a heterologous vaccination scheme. Aside from using a different vaccine platform altogether, two LAVs could be used, each based on a different SARS-CoV-2 variant with poor antibody cross-reactivity. In the end, this may not be a major issue if the immunity is sufficiently strong after the first dose, if it is sufficiently long-lasting or if the booster dose is administered once the immunity wanes below the levels that significantly restrict replication.

### 4.4. Limitations and Implications

Finally, according to the limitations and implications of this review, it must be stated that attenuating mutations may have different effects in different coronaviruses even if the amino acid residues they affect are part of a clearly conserved sequence. Several examples of this are known in the literature; for example, NSP14 proof-reading exonuclease is redundant for replication but necessary for virulence in SARS-CoV or MHV [183,184]. On the contrary, it is not dispensable in SARS-CoV-2 and MERS-CoV [157]. E protein deletion in SARS-CoV reduces virulence and cell culture titers, but it leads to a packaging-deficient replicon in the cases of MERS-CoV or TGEV [83,84,85,86,87,88,89,90,91,92,93,94,185]. Additional examples are presented in Appendix B. Therefore, it is clear that attenuating mutations may not work as expected in the novel virus if they were only tested in related viruses. Several strategies must be tested in parallel to account for potential failures if reverse genetics is used to apply attenuating mutations in a novel pathogen to create a vaccine. An additional problem is simply that the results of animal studies may not translate well to humans. It is also certain that a publication bias exists because there is a mention of codon-deoptimized SARS-CoV in literature based on a personal communication [93] and human clinical trial data of one attenuated SARS-CoV-2 virus have been presented at a conference [186]; a full report is not available for either of those studies.

Since no attenuated COVID-19 vaccine is currently marketed, the results from clinical trials are not available and cannot be discussed. Instead, it seems clear that the potential of attenuated SARS-CoV-2 as a vaccine was largely neglected. Although, at this point, a new COVID-19 vaccine may not have a significant impact, it may be worth it if the development of LAVs against emerging pathogens would receive higher priority in the future. Perhaps a range of attenuating mutations may be identified in different viral taxa to facilitate the rapid development of LAVs through reverse genetics. Nevertheless, if another pandemic coronavirus emerges [187,188,189], many mutations identified in this review may still be applicable.

## 5. Conclusions

In conclusion, attenuated COVID-19 vaccines had considerable potential to alter the course of the COVID-19 pandemic. Animal studies have demonstrated the potential for the safety and efficacy of attenuated SARS-CoV-2 vaccines with the additional benefits of being one-dose and needle-free, allowing for intranasal administration and reducing transmission. Attenuated SARS-CoV-2 would likely cause transient, asymptomatic pneumonia at most. FCS disruption seems to be the best strategy for achieving SARS-CoV-2 attenuation because it was proven to be hard to revert, transmission-preventing and immunogenicity non-altering, while maintaining high titer and stability in Vero E6 cells. Another promising strategy is TRS replacement, which has the potential to prevent recombination between the vaccine and WT viruses. Other strategies are usually aimed at preventing the evasion of innate immunity by the virus. A combination of attenuation methods to achieve a vaccine virus that is safe, immunogenic and does not revert is probably the most desired. Reverse genetics seems to be the method of choice for obtaining an attenuated COVID-19 vaccine because of its predictable results. SARS-CoV-2 passaged in cell culture usually loses FCS, which is a result that was also achieved through reverse genetics. Other mutations may also arise in them, the exact importance of which was not thoroughly studied. Notably, the recently emerged and highly infectious SARS-CoV-2 variant of concern, Omicron, may be perceived as a weakly attenuated SARS-CoV-2 virus.

## Data Availability

The data presented in this study are available in the article and Supplementary Materials. Due to the large volume, raw data extracted from studies are available on reasonable request.

## Supplementary Materials

The following supporting information can be downloaded at: https://www.mdpi.com/article/10.3390/v14050991/s1, Supplementary Materials S1: Search keys utilized in database searches, Table S1: A complete list of GenBank entries that were used to assess the applicability of mutations in this article, Table S2a: A complete list of viruses included in this review, Table S2b: A summary of the results of in vivo experiments involving the included viruses excluding experiments involving knock-out animal models, Table S2c: A summary of the results of experiments that aimed to study the reversion to virulence of attenuated coronaviruses in vitro, Table S2d: A summary of the results of experiments that aimed to study immunogenicity of attenuated coronaviruses, Table S2e: A summary of the results of the challenge studies, Table S3: PRISMA 2020 Checklist, Figure S1a–S1af: Sequence alignments of the conserved coronaviral amino acid sequences with attenuation targets identified in this review, Figure S2: PRISMA 2020 flow diagram of studies, Table S4: A complete list of database search results with reasons for exclusion.

## Appendix A

The remaining PRISMA 2020 criteria are elaborated upon here. Planned synthesis of the results was judged impossible, as the animal trials were very heterogeneous. Due to the qualitative nature of this review, missing data were not considered an issue. Risk of Bias assessment was not performed due to the reporting of animal studies being less thorough than human trials. Conflicts of interest were not assessed, as most of the included studies did not provide directly commercially relevant results.

## Appendix B

Some additional examples of mutations in conserved amino acid residues causing different phenotypes in coronaviruses have been found. A SARS-CoV NSP14 D331A mutant is not viable, while for MERS-CoV, this mutation is severely crippling [158]. The corresponding D330A mutation in MHV has little effect in vitro. In MHV-A59, Y414A substitution in NSP14 is tolerated but attenuating in vivo, although such a mutant was not viable in the case of MHV-JHM. This was attributed to the less robust RNA synthesis of MHV-JHM [158]. In the case of MHV, the cleavage site between NSP10 and NSP11/12 may be disrupted, but such an alteration leads to a non-viable virus in the case of IBV. In IBV, the lack of a proteolytic cleavage site between NSP9 and NSP10 leads to an attenuated in vitro but viable virus, but such a mutation is lethal in MHV [190,191]. Stobart et al. [192] constructed chimeric MHV viruses with NSP5 proteases derived from HCoV-HKU1 and OC43. S133A, V148A and F219L NSP5 mutations, which are responsible for the temperature sensitivity of some MHV-A59 viruses, were then introduced into those chimeras. Only some mutants were recovered, and out of those, only MHV-OC43 chimera with S133A mutation was temperature-sensitive.
