# Supplementary material for: The Reassessed Potential of SARS-CoV-2 Attenuation for COVID-19 Vaccine Development—A Systematic Review
Source: Viruses. 2022 May 7;14(5):991. doi: 10.3390/v14050991 (PMC9146402; doi:10.3390/v14050991)
Supplement: Supplementary file 1 [file viruses-14-00991-s001.zip › Supplementary Figure S2.pdf]

PRISMA 2020 flow diagram for new systematic reviews which included searches of databases, registers and other sources

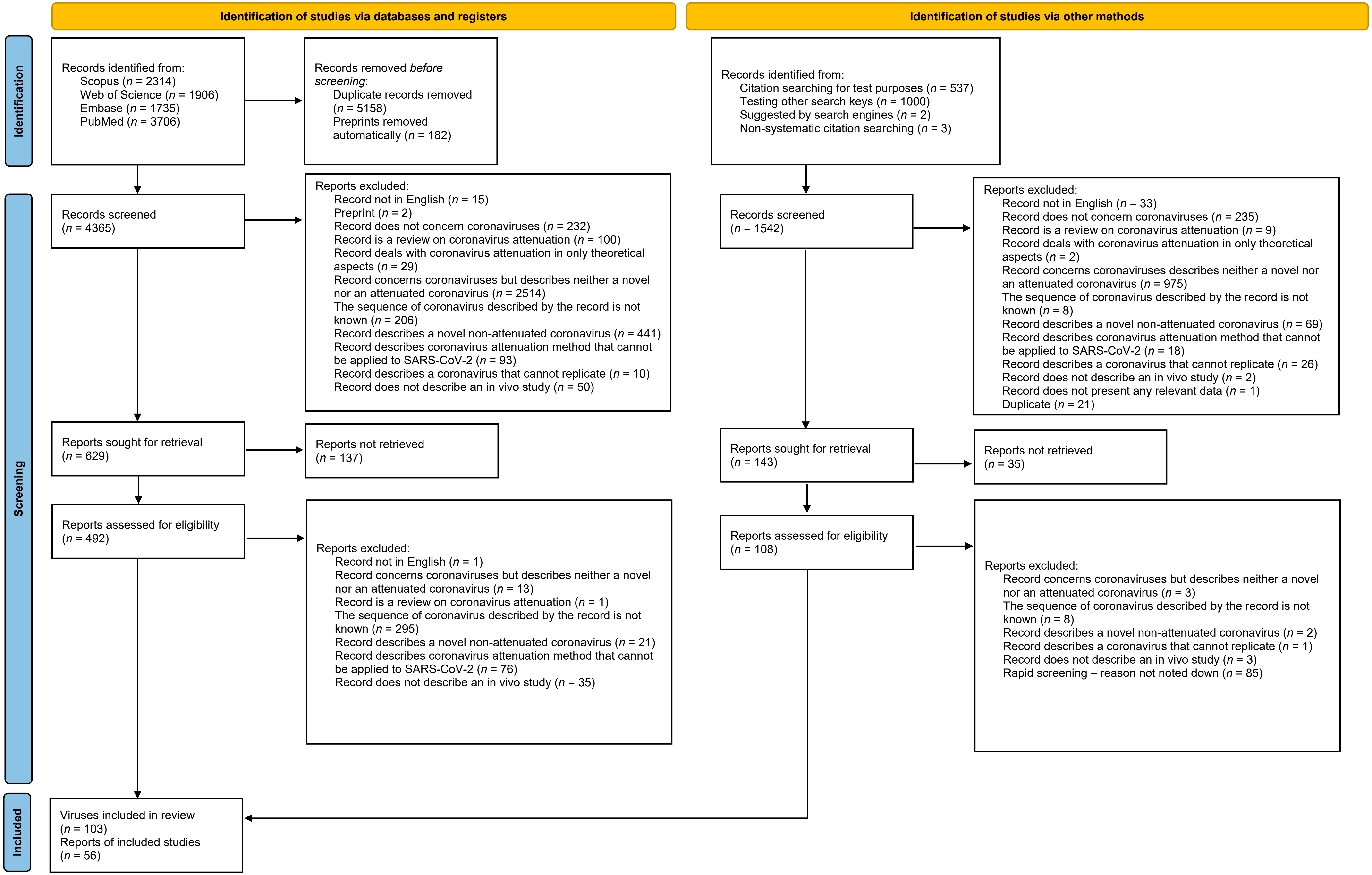

Figure S2. PRISMA 2020 flow diagram of studies.

*Consider, if feasible to do so, reporting the number of records identified from each database or register searched (rather than the total number across all databases/registers).
**If automation tools were used, indicate how many records were excluded by a human and how many were excluded by automation tools.

From: Page MJ, McKenzie JE, Bossuyt PM, Boutron I, Hoffmann TC, Mulrow CD, et al. The PRISMA 2020 statement: an updated guideline for reporting systematic reviews. BMJ 2021;372:n71. doi: 10.1136/bmj.n71. For more information, visit: http://www.prisma-statement.org/
