# Supplementary material for: The Reassessed Potential of SARS-CoV-2 Attenuation for COVID-19 Vaccine Development—A Systematic Review
Source: Viruses. 2022 May 7;14(5):991. doi: 10.3390/v14050991 (PMC9146402; doi:10.3390/v14050991)
Supplement: Supplementary file 1 [file viruses-14-00991-s001.zip › Supplementary Figures S1a-S1af.pdf]

Supplementary Figures S1a-S1af: Sequence alignments of the conserved coronaviral aminoacid sequences with attenuation targets identified in this review.

The WT sequence of the SARS-CoV-2 protein (top) is compared against the sequence of another WT coronavirus(es) (bottom) and the amino acid residues known to be altered in the attenuated non-SARS-CoV-2 virus are indicated. The exact mutation position is presented for both SARS-CoV-2 (top) and other coronaviruses (bottom).

Virus	GenBank entry	Protein	Sequence
			S3A V5L T9A T11A N15A
SARS-CoV-2 WuhanHu-1	YP_009724392.1	Envelope	1 M Y S F V S E E T G T L I V N S V L L F 20
SARS-CoV MA15	ACZ72213.1	Envelope	1 M Y S F V S E E T G T L I V N S V L L F 20
			S3A V5L T9A T11A N15A

Figure S1a. A sequence alignment of the Envelope protein sequences with attenuating mutations indicated – Part 1.

Virus	GenBank entry	Protein	Sequence
			N15A V25F
SARS-CoV-2 Wuhan-Hu-1	YP_009724392.1	Envelope	15 N S V L L F L A F V V F L L V T L A 32
SARS-CoV MA15	ACZ72213.1	Envelope	15 N S V L L F L A F V V F L L V T L A 32
			N15A V25F

Figure S1b. A sequence alignment of the Envelope protein sequences with attenuating mutations indicated – Part 2.

Virus	GenBank entry	Protein	Sequence
			Δ38-45 Δ46-52
SARS-CoV-2 Wuhan-Hu-1	YP_009724392.1	Envelope	34 L T A L R L C A Y C C N I V N V S L V K P S F Y 57
SARS-CoV MA15	ACZ72213.1	Envelope	34 L T A L R L C A Y C C N I V N V S L V K P T V Y 57
			Δ38-45 Δ46-52

Figure S1c. A sequence alignment of the Envelope protein sequences with attenuating mutations indicated – Part 3.

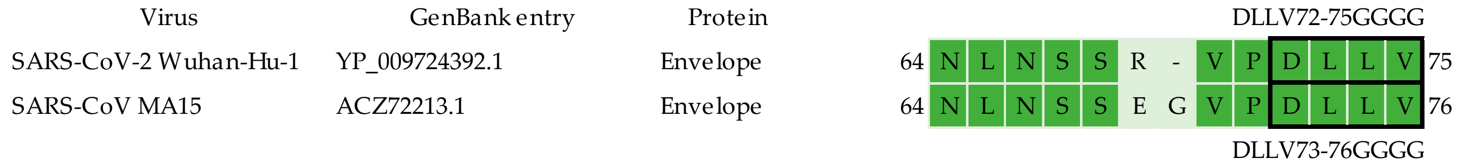

Figure S1d. A sequence alignment of the Envelope protein sequences with attenuating mutations indicated – Part 4.

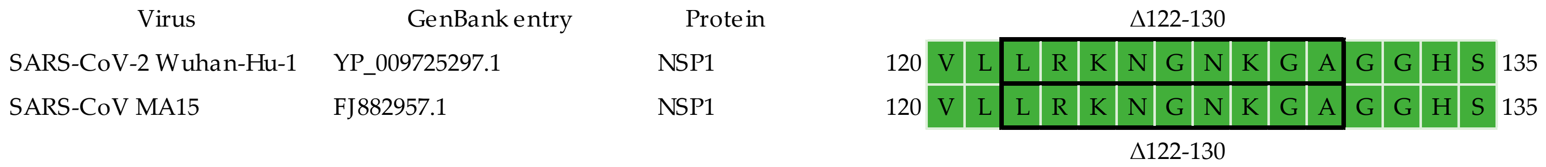

Figure S1e. A sequence alignment of the NSP1 sequences with attenuating mutations indicated – Part 1.

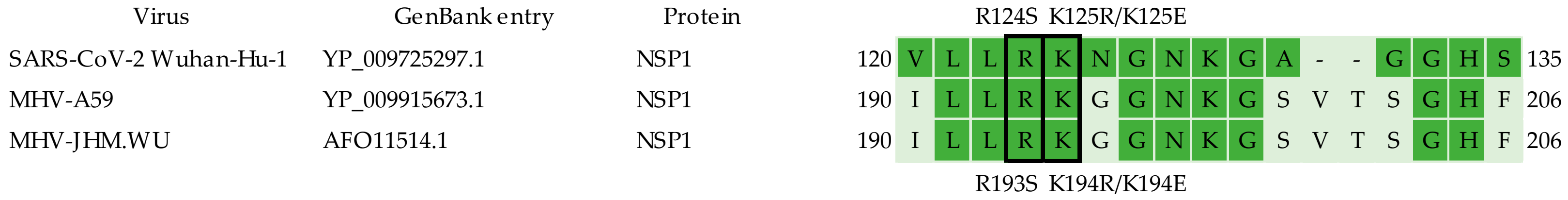

Figure S1f. A sequence alignment of the NSP1 sequences with attenuating mutations indicated – Part 2.

F480L

Virus	GenBank entry	Protein	Start	Sequence	End
SARS-CoV-2 Wuhan-Hu-1	YP_009725307.1	NSP12	470	L F V V E V V D K Y F D C Y D G G C I N A	490
SARS-CoV MA15 NanoLuc	ACZ72209.1	NSP12	470	L F V V E V V D K Y F D C Y D G G C I N A	490
MHV-A59	YP_009924352.1	NSP12	466	L F V L E V V N K Y F E I Y E G G C I P A	486

F480L/F476L

Figure S1g. A sequence alignment of the NSP12 sequences with attenuating mutations indicated – Part 1.

V557L

Virus	GenBank entry	Protein	Start	Sequence	End
SARS-CoV-2 Wuhan-Hu-1	YP_009725307.1	NSP12	547	A I S A K N R A R T V A G V S I C S T M T	567
SARS-CoV MA15 NanoLuc	ACZ72209.1	NSP12	547	A I S A K N R A R T V A G V S I C S T M T	567
MHV-A59	YP_009924352.1	NSP12	543	A I S A K N R A R T V A G V S I L S T M T	563

V557L/V553L

Figure S1h. A sequence alignment of the NSP12 sequences with attenuating mutations indicated – Part 2.

A336V

Virus	GenBank entry	Protein	Start	Sequence	End
SARS-CoV-2 Wuhan-Hu-1	YP_009725307.1	NSP13	325	P I D K C S R I I P A R A R V E C F D K F	346
MHV-JHM.WU	AFO11514.1	NSP13	324	N I N D C T R I V P A K V R V D C Y D K F	345

A335V

Figure S1i. A sequence alignment of the NSP13 sequences with attenuating mutations indicated.

Virus	GenBank entry	Protein		D331A	
SARS-CoV-2 Wuhan-Hu-1	YP_009725309.1	NSP14	321	L L A D K F P V L H D I G N P K A I K C V	341
MHV-A59	YP_009924354.1	NSP14	320	M L C N R Y D V C Y D I G N P K G L A C V	340
				D330A	

Figure S1j. A sequence alignment of the NSP14 sequences with attenuating mutations indicated – Part 1.

Virus	GenBank entry	Protein		N386A / V398L	
SARS-CoV-2 Wuhan-Hu-1	YP_009725309.1	NSP14	380	G V C L F W N C N V D R Y P A N S I V C R F D T R V	405
MHV-A59	YP_009924354.1	NSP14	374	G L C M F W N C N V D K Y P A N A V V C R F D T R V	399
M41R-nsp10.14rep	AAW33784.1	NSP14	375	G L C M F W N C N V D C Y P D N S L V C R Y D T R N	400
				N380A / V393L	

Figure S1k. A sequence alignment of the NSP14 sequences with attenuating mutations indicated – Part 2.

Virus	GenBank entry	Protein		Y420H/Y420A	
SARS-CoV-2 Wuhan-Hu-1	YP_009725309.1	NSP14	410	N L P G C N G G S L Y V N K H A F H T S P	430
MHV-A59	YP_009924354.1	NSP14	404	N L P G C D G G S L Y V N K H A F H T P A	424
				Y414H/Y414A	

Figure S1l. A sequence alignment of the NSP14 sequences with attenuating mutations indicated – Part 3.

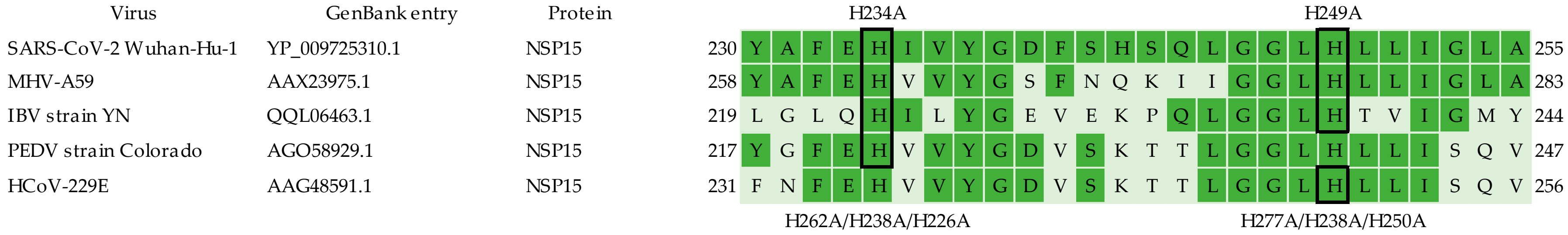

Figure S1m. A sequence alignment of the NSP15 sequences with attenuating mutations indicated – Part 1.

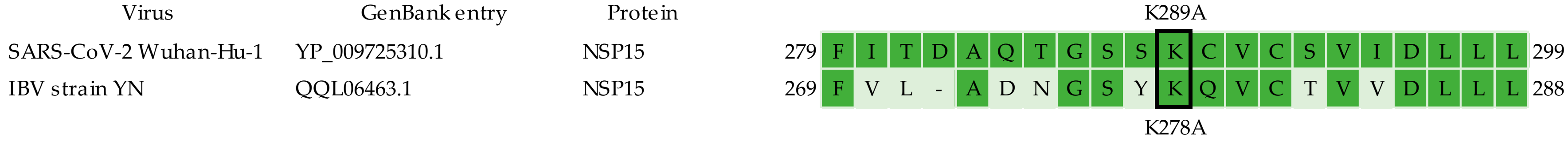

Figure S1n. A sequence alignment of the NSP15 sequences with attenuating mutations indicated – Part 2.

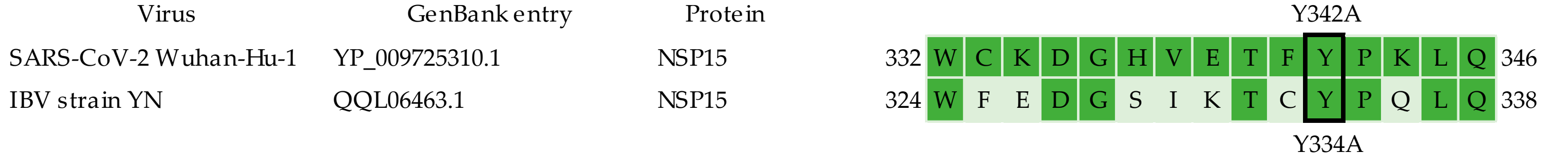

Figure S1o. A sequence alignment of the NSP15 sequences with attenuating mutations indicated – Part 3.

Y15A

Virus	GenBank entry	Protein		Sequence	
SARS-CoV-2 Wuhan-Hu-1	YP_009725311.1	NSP16	5	W Q P G V A M P N L Y K M Q R M L L E K C	25
MHV-A59	YP_009924356.1	NSP16	5	W K P G Y V M P V L Y K Y L E S P L E R V	25

Y15A

Figure S1p. A sequence alignment of the NSP16 sequences with attenuating mutations indicated – Part 1.

D130A

Virus	GenBank entry	Protein		Sequence	
SARS-CoV-2 Wuhan-Hu-1	YP_009725311.1	NSP16	120	T A N K W D L I I S D M Y D P K T K N V T	140
SARS-CoV Urbani	AAP13442.1	NSP16	120	T A N K W D L I I S D M Y D P R T K H V T	140
SARS-CoV MA15	ACZ72209.1	NSP16	120	T A N K W D L I I S D M Y D P R T K H V T	140
HCoV-229E	AAG48591.1	NSP16	119	L E D K F D L L I S D M Y D G R T K A I D	139
PEDV icPC22A	ARM65495.1	NSP16	119	L S D K F D L V I S D M Y D G K I K S C D	139
MERS-CoV EMC	YP_009047227.1	NSP16	120	V G Q Q V D L V I S D M Y D P T T K N V T	140
MERS-CoV MA1	JX869059.2	NSP16	120	V G Q Q V D L V I S D M Y D P T T K N V T	140

D130A/D129A

Figure S1r. A sequence alignment of the NSP16 sequences with attenuating mutations indicated – Part 2.

K170A

Virus	GenBank entry	Protein		Sequence	
SARS-CoV-2 Wuhan-Hu-1	YP_009725311.1	NSP16	160	K L A L G G S V A I K I T E H S W N A D L	180
SARS-CoV Urbani	AAP13442.1	NSP16	160	K L A L G G S I A V K I T E H S W N A D L	180
PEDV icPC22A	ARM65495.1	NSP16	159	K L A L G G T V A I K V T E F S W N K K L	179

K170A/K169A

Figure S1s. A sequence alignment of the NSP16 sequences with attenuating mutations indicated – Part 3.

K46A

Virus	GenBank entry	Protein																							
SARS-CoV-2 Wuhan-Hu-1	YP_009725311.1	NSP16	36	L	P	K	G	I	M	M	N	V	A	K	Y	T	Q	L	C	Q	Y	L	N	T	56
SARS-CoV Urbani	AAP13442.1	NSP16	36	I	P	K	G	I	M	M	N	V	A	K	Y	T	Q	L	C	Q	Y	L	N	T	56
PEDV icPC22A	ARM65495.1	NSP16	35	L	P	D	G	I	M	F	N	V	V	K	Y	T	Q	L	C	Q	Y	L	N	S	55

K46A/K45A

Figure S1t. A sequence alignment of the NSP16 sequences with attenuating mutations indicated – Part 4.

E203A

Virus	GenBank entry	Protein																							
SARS-CoV-2 Wuhan-Hu-1	YP_009725311.1	NSP16	193	F	V	T	N	V	N	A	S	S	S	E	A	F	L	I	G	C	N	Y	L	G	213
PEDV icPC22A	ARM65495.1	NSP16	192	F	C	T	S	V	N	T	S	S	S	E	A	F	L	I	G	V	H	Y	L	G	212

E202A

Figure S1u. A sequence alignment of the NSP16 sequences with attenuating mutations indicated – Part 5.

D226A

Virus	GenBank entry	Protein																							
SARS-CoV-2 Wuhan-Hu-1	YP_009725299.1	NSP3	216	L	T	D	N	V	Y	I	K	N	A	D	I	V	E	E	A	K	K	V	K	P	236
SARS-CoV MA15	ACZ72208.1	NSP3	194	L	T	D	N	V	A	I	K	C	V	D	I	V	K	E	A	Q	S	A	N	P	214
rJIA-GFPrevN1347	YP_209246.1	NSP3	487	I	T	P	N	V	C	F	V	K	G	D	V	I	K	V	L	R	R	V	G	A	507

D204A/D497A

Figure S1v. A sequence alignment of the NSP3 sequences with attenuating mutations indicated – Part 1.

Virus	GenBank entry	Protein		N244A H249A	
SARS-CoV-2 Wuhan-Hu-1	YP_009725299.1	NSP3	234	V K P T V V V N A A N V Y L K H G G G V A G A L N K	259
SARS-CoV MA15	ACZ72208.1	NSP3	212	A N P M V I V N A A N I H L K H G G G V A G A L N K	237
rJIA-GFPrevN1347	YP_209246.1	NSP3	505	V G A E V I V N P A N G R M A H G A G V A G A I A K	530
MHV-A59	AAX23975.1	NSP3	506	V N A E V I V N P A N G R M A H G A G V A G A I A E	531
				N222A/N515A/N516A H227A	

Figure S1w. A sequence alignment of the NSP3 sequences with attenuating mutations indicated – Part 2.

Virus	GenBank entry	Protein		A333G G334V	
SARS-CoV-2 Wuhan-Hu-1	YP_009725299.1	NSP3	323	H E V L L A P L L S A G I F G A D P I H S L	344
SARS-CoV MA15	ACZ72208.1	NSP3	301	Q D I L L A P L L S A G I F G A K P L Q S L	322
rJIA-GFPrevN1347	YP_209246.1	NSP3	596	C D N V V T T L I S A G I F S V P T D V S L	617
				A606G G312V/G607V	

Figure S1x. A sequence alignment of the NSP3 sequences with attenuating mutations indicated – Part 3.

Virus	GenBank entry	Protein		S40A	
SARS-CoV-2 Wuhan-Hu-1	YP_009724391.1	ORF3a	1	R A T A T I P I Q A S L P F G W L I V G V	32
SARS-CoV MA15	ACZ72211.1	ORF3a	1	H A T A T I P L Q A S L P F G W L V I G V	32
				S40A	

Figure S1y. A sequence alignment of the ORF3a protein sequences with attenuating mutations indicated – Part 1.

Virus	GenBank entry	Protein	S58A
SARS-CoV-2 Wuhan-Hu-1	YP_009724391.1	ORF3a	48 I G V A F L A V F Q [S] A T K I I A L N K R 68
SARS-CoV MA15	ACZ72211.1	ORF3a	48 V G V A L L A V F Q [S] A S K I I T L K K R 68
			S58A

Figure S1z. A sequence alignment of the ORF3a protein sequences with attenuating mutations indicated – Part 2.

Virus	GenBank entry	Protein	Y109A, Y113A, Q116A
SARS-CoV-2 Wuhan-Hu-1	YP_009724391.1	ORF3a	104 P F L Y L [Y] A L V [Y] F L [Q] S I N F V 121
SARS-CoV MA15	ACZ72211.1	ORF3a	104 Q F L Y L [Y] A L I [Y] F L [Q] C I N A C 121
			Y109A, Y113A, Q116A

Figure S1aa. A sequence alignment of the ORF3a protein sequences with attenuating mutations indicated – Part 3.

Virus	GenBank entry	Protein	SVPL272-275GMSM
SARS-CoV-2 Wuhan-Hu-1	YP_009724391.1	ORF3a	262 P I Y D E P T T T T [S V P L] 275
SARS-CoV MA15	ACZ72211.1	ORF3a	261 P I Y D E P T T T T [S V P L] 274
			SVPL271-274GMSM

Figure S1ab. A sequence alignment of the ORF3a protein sequences with attenuating mutations indicated – Part 3.

Virus	GenBank entry	Protein	T345I
SARS-CoV-2 Wuhan-Hu-1	YP_009724390.1	Spike	335 L C P F G E V F N A [T] R F A S V Y A W N R 355
SARS-CoV Urbani	AAP13441.1	Spike	332 L C P F G E V F N A [T] K F P S V Y A W E R 342
			T332I

Figure S1ac. A sequence alignment of the Spike protein sequences with attenuating mutations indicated – Part 1.

Q965H

Virus	GenBank entry	Protein			
SARS-CoV-2 Wuhan-Hu-1	YP_009724390.1	Spike	955	N A Q A L N T L V K Q L S S N F G A I S S	975
SJHM-RA59/S4R29	YP_209233.1	Spike	1057	N A E A L N N L L N Q L S N R F G A I S A	1077

Q1067H

Figure S1ad. A sequence alignment of the Spike protein sequences with attenuating mutations indicated – Part 2.

Q992H

Virus	GenBank entry	Protein			
SARS-CoV-2 Wuhan-Hu-1	YP_009724390.1	Spike	982	S R L D K V E A E V Q I D R L I T G R L Q	1002
SJHM-RA59/S4R29	YP_209233.1	Spike	1084	T R L D A V E A K A Q I D R L I N G R L T	1104

Q1094H

Figure S1ae. A sequence alignment of the Spike protein sequences with attenuating mutations indicated – Part 3.

L1012R

Virus	GenBank entry	Protein			
SARS-CoV-2 Wuhan-Hu-1	YP_009724390.1	Spike	982	Q S L Q T Y V T Q Q L I R A A E I R A S A	1002
SJHM-RA59/S4R29	YP_209233.1	Spike	1084	T A L N A Y I S K Q L S D S T L I K F S A	1104

L1114R

Figure S1af. A sequence alignment of the Spike protein sequences with attenuating mutations indicated – Part 4.
