# Supplementary material for: The Reassessed Potential of SARS-CoV-2 Attenuation for COVID-19 Vaccine Development—A Systematic Review
Source: Viruses. 2022 May 7;14(5):991. doi: 10.3390/v14050991 (PMC9146402; doi:10.3390/v14050991)
Supplement: Supplementary file 1 [file viruses-14-00991-s001.zip › Supplementary Material S1.pdf]

Supplementary Material 1

Search keys utilized in database searches

PubMed

(coronavirus*[Title] OR alphacoronavirus*[Title] OR betacoronavirus*[Title] OR gammacoronavirus*[Title] OR deltacoronavirus*[Title] OR SARS[Title] OR MERS[Title] OR PEDV[Title] OR TGEV[Title] OR COV[Title] OR hCoV [Title] OR nCoV[Title] OR IBV[Title] OR PDCoV[Title] OR PRCV[Title] OR FCoV[Title] OR MHV[Title] OR (severe[Title] AND acute[Title] AND respiratory[Title]) OR (middle[Title] AND east*[Title] AND respiratory[Title]) OR ((porcine[Title] OR pig[Title]) AND diarrhea[Title] AND (virus[Title]OR viral[Title])) OR (transmissible[Title] AND gastroenteritis[Title]) OR (infectious[Title] AND bronchitis[Title]) OR ((feline[Title] OR infectious[Title]) AND peritonitis[Title]) OR ((murine[Title] OR mouse[Title]) AND hepatitis[Title]) OR (covid[Title] AND attenuat*[Title] AND vaccin*[Title]) OR OC43[Title] OR HKU1[Title] OR NL63[Title] OR 229E[Title] OR colacovirus[Title] OR decacovirus[Title] OR duvinavirus[Title] OR luchacovirus[Title] OR minacovirus[Title] OR minunacovirus[Title] OR myotacovirus[Title] OR nyctacovirus[Title] OR pedacovirus[Title] OR rhinacovirus[Title] OR setracovirus[Title] OR soracovirus[Title] OR sunacovirus[Title] OR tegacovirus[Title] OR embecovirus[Title] OR hibecovirus[Title] OR merbecovirus[Title] OR nobecovirus[Title] OR sarbecovirus[Title] OR andecovirus[Title] OR buldecovirus[Title] OR herdecovirus[Title] OR brangacovirus[Title] OR cegacovirus[Title] OR igacovirus[Title] OR CDPHE15[Title] OR HKU10[Title] OR "HuB-2013"[Title] OR HKU8[Title] OR 3398[Title] OR HKU2[Title] OR BtKYNL63[Title] OR T14[Title] OR X74[Title] OR HKU24[Title] OR 2JL14[Title] OR Zheijang2013[Title] OR HKU5[Title] OR HKU4[Title] OR C704[Title] OR GCCDC1[Title] OR HKU9[Title] OR HKU20[Title] OR HKU11[Title] OR HKU21[Title] OR HKU15[Title] OR HKU13[Title] OR HKU16[Title] OR HKU19[Title] OR CB17[Title] OR SW1[Title] OR 9203[Title] OR 2714[Title] OR "HuPn-2018" [Title] OR WIV1[Title] OR RacCS203[Title] OR RmYN02[Title] OR RpYN06[Title] OR SHC014[Title]) AND (attenuat*[tiab] OR attenuated vaccines[mh] OR (temperature[tiab] AND sensitiv*[tiab]) OR (live[tiab] AND (vaccin*[tiab] OR inocul*[tiab])) OR ((cold[tiab] OR temperature[tiab] OR cell[tiab] OR culture[tiab]) AND adapt*[tiab]) OR avirulent[tiab] OR ("non-virulent"[tiab]) OR ((vaccin*[tiab] or inocul*[tiab]) AND strain*[tiab])) NOT (review[sb])

Scopus

(TITLE(*coronavirus OR {SARS} OR {MERS} OR PEDV OR TGEV OR COV OR hCoV OR nCoV OR IBV OR PDCoV OR PRCV OR FCoV OR MHV OR (severe AND acute AND respiratory) OR (middle AND east* AND respiratory) OR ((porcine OR pig) AND diarrhea AND (virus OR viral)) OR (transmissible AND gastroenteritis) OR (infectious AND bronchitis) OR ((feline OR infectious) AND peritonitis) OR ((murine OR mouse) AND hepatitis) OR OC43 OR HKU1 OR NL63 OR 229E OR (covid AND attenuat* AND vaccin*) OR colacovirus OR decacovirus OR duvinavirus OR luchacovirus OR minacovirus OR minunacovirus OR myotacovirus OR nyctacovirus OR pedacovirus OR rhinacovirus OR setracovirus OR soracovirus OR sunacovirus OR tegacovirus OR embecovirus OR hibecovirus OR merbecovirus OR nobecovirus OR sarbecovirus OR andecovirus OR buldecovirus OR herdecovirus OR brangacovirus OR cegacovirus OR igacovirus OR CDPHE15 OR HKU OR HKU? OR HKU?? OR {HuB-2013} OR 3398 OR BtKYNL63 OR T14 OR X74 OR 2JL14 OR Zheijang2013 OR C704 OR GCCDC1 OR HKU11 OR CB17 OR SW1 OR 9203 OR 2714 OR {HuPn-2018} OR WIV1 OR RacCS203 OR RmYN02 OR RpYN06 OR SHC014) AND TITLE-ABS-KEY(attenuat* OR (temperature W/2 sensitiv*) OR (live W/5 (vaccin* OR inocul*)) OR ((cold OR temperature OR cell OR

culture) W/3 adapt*) OR avirulent OR (non Pre/0 virulent) OR ((vaccin* OR inocul*) W/5 strain*))) AND NOT DOCTYPE(RE)

Web of Science

((TI=(*coronavirus OR “SARS” OR “MERS” OR PEDV OR TGEV OR COV OR IBV OR PDCoV OR PRCV OR FCoV OR MHV OR nCoV OR hCoV OR (severe AND acute AND respiratory) OR (middle AND east* AND respiratory) OR ((porcine OR pig) AND diarrhea AND (virus OR viral)) OR (transmissible AND gastroenteritis) OR (infectious AND bronchitis) OR ((feline OR infectious) AND peritonitis) OR ((murine OR mouse) AND hepatitis) OR OC43 OR HKU1 OR NL63 OR 229E OR (covid AND attenuat* AND vaccin*) colacovirus OR decacovirus OR duvinavirus OR luchacovirus OR minacovirus OR minunacovirus OR myotacovirus OR nyctacovirus OR pedacovirus OR rhinacovirus OR setracovirus OR soracovirus OR sunacovirus OR tegacovirus OR embecovirus OR hibecovirus OR merbecovirus OR nobecovirus OR sarbecovirus OR andecovirus OR buldecovirus OR herdecovirus OR brangacovirus OR cegacovirus OR igacovirus OR CDPHE15 OR HKU?$ OR HuB-2013 OR 3398 OR BtKYNL63 OR T14 OR X74 OR 2JL14 OR Zheijang2013 OR C704 OR GCCDC1 OR CB17 OR SW1 OR 9203 OR 2714 OR HuPn-2018 OR WIV1 OR RacCS203 OR RmYN02 OR RpYN06 OR SHC014)) AND ((AB=(attenuat* OR (temperature NEAR/2 sensitiv*) OR (live NEAR/5 (vaccin* OR inocul*)) OR ((cold OR temperature OR cell OR culture) NEAR/3 adapt*) OR avirulent OR (non NEAR/0 virulent) OR (strain NEAR/5 (vaccin* OR inocul*)))) OR (TI=(attenuat* OR (temperature NEAR/2 sensitiv*) OR (live NEAR/5 (vaccin* OR inocul*)) OR ((cold OR temperature OR cell OR culture) NEAR/3 adapt*) OR avirulent OR (non NEAR/0 virulent) OR (strain NEAR/5 (vaccin* OR inocul*)))) OR (AK=(attenuat* OR (temperature NEAR/2 sensitiv*) OR (live NEAR/5 (vaccin* OR inocul*)) OR ((cold OR temperature OR cell OR culture) NEAR/3 adapt*) OR avirulent OR (non NEAR/0 virulent) OR (strain NEAR/5 (vaccin* OR inocul*)))) OR (KP=(attenuat* OR (temperature NEAR/2 sensitiv*) OR (live NEAR/5 (vaccin* OR inocul*)) OR ((cold OR temperature OR cell OR culture) NEAR/3 adapt*) OR avirulent OR (non NEAR/0 virulent) OR (strain NEAR/5 (vaccin* OR inocul*)))))) NOT DT=(Review)

Embase

(coronavirus* OR alphacoronavirus* OR betacoronavirus* OR gammacoronavirus* OR deltacoronavirus* OR SARS OR MERS OR PEDV OR TGEV OR COV OR IBV OR PDCoV OR PRCV OR FCoV OR MHV OR hCoV OR nCoV OR (severe AND acute AND respiratory) OR (middle AND east* AND respiratory) OR ((porcine OR pig*) AND diarrhea AND (virus* OR viral)) OR (transmissible AND gastroenteritis) OR (infectious AND bronchitis) OR (feline AND peritonitis) OR ((murine OR mouse) AND hepatitis) OR OC43 OR HKU1 OR NL63 OR 229E OR (covid AND attenuat* AND vaccin*) OR colacovirus* OR decacovirus* OR duvinavirus* OR luchacovirus* OR minacovirus* OR minunacovirus* OR myotacovirus* OR nyctacovirus* OR pedacovirus* OR rhinacovirus* OR setracovirus* OR soracovirus* OR sunacovirus* OR tegacovirus* OR embecovirus* OR hibecovirus* OR merbecovirus OR nobecovirus OR sarbecovirus OR andecovirus OR buldecovirus OR herdecovirus OR brangacovirus OR cegacovirus OR igacovirus OR CDPHE15 OR HuB-2013 OR 3398 OR BtKYNL63 OR T14 OR X74 OR 2JL14 OR Zheijang2013 OR HKU?$ OR C704 OR GCCDC1 OR CB17 OR SW1 OR 9203 OR 2714 OR HuPn-2018 OR WIV1 OR RacCS203 OR RmYN02 OR RpYN06 OR SHC014):ti AND (attenuat* OR (temperature NEAR/2 sensitiv*) OR (live NEAR/5 (vaccin* OR inocul*)) OR ((cold OR temperature OR cell OR culture) NEAR/3 adapt*) OR avirulent OR (non NEXT/0 virulent) OR ((vaccin* OR inocul*) NEAR/3 strain*)):ti,ab,kw NOT review:it
