# Supplementary material for: The Reassessed Potential of SARS-CoV-2 Attenuation for COVID-19 Vaccine Development—A Systematic Review
Source: Viruses. 2022 May 7;14(5):991. doi: 10.3390/v14050991 (PMC9146402; doi:10.3390/v14050991)
Supplement: Supplementary file 1 [file viruses-14-00991-s001.zip › Supplementary Tables S2a-S2e.pdf]

The reassessed potential of SARS-CoV-2 attenuation for COVID-19 vaccine development - a systematic review

Marcin Goławski, Piotr Lewandowski, Iwona Jabłońska and Marcin Delijewski

Supplementary Material; Tables S2a-S2e

Table S2a. A complete list of viruses included in this review.

Parental strain	Known as	Mutations	Corresponding mutations in SARS-CoV-2	Origins	Reference
SARS-CoV-2	strain TY38-873 (Omicron)<br>Lineage BA.1	NSP3: K38R P985L T1004I SL1265–1266I A1892T NSP4 T492I<br>NSP5 P132Y<br>NSP6: Δ105–107 I189V NSP12 P303L<br>NSP14 I42V<br>Spike: A67V Δ69–70 T95I G142D Δ143-145 NL211–212I RD214–215EPE G339D S371L S373P S375F K417N N440K G446S S477N T478K E484A Q493R G496S Q498R N501Y Y505H T547K D614G H655Y N679K P681H N764K D796Y N856K Q954H N969K L981F<br>Envelope T9I<br>Membrane: D3G Q19E A63T<br>Nucleocapsid: P13L Δ31–33 RG203–204KR<br>ORF10 V30L	-	Naturally occurring	[1]
SARS-CoV-2	Omicron variant, strain B.1.1.529, isolate hCoV-19/USA/WI-WSLH-221686/2021<br>Lineage BA.1	NSP3: K38R SL1265–1266I A1892T<br>NSP4 T492I NSP5 P132H<br>NSP6 Δ106-108<br>NSP12 P323L<br>NSP14 I42V<br>NSP16 L126F<br>Spike: A67V Δ69–70 T95I G142D Δ143-145 NL211-212I 214EPE insertion G339D S371F S373P S375F K417N N440K G446S S477N T478K E484A Q493R G496R Q498R N501Y Y505H T547K D614G H655Y N679K P681H D796Y N856K Q954H N969K	-	Naturally occurring	[2]

		L981F Envelope: T9I<br>Membrane: D3G Q19E A63T<br>Nucleocapsid: P13L Δ31–33 RG203–204KR			
SARS-CoV-2	hCoV-19/USA/NY-MSHSPSP-PV44476/2021<br>Lineage BA.1	NSP3: K38R SL1265–1266I A1892T<br>NSP4 T492I NSP5 P132H NSP6 Δ106–108<br>NSP12 P323L<br>NSP14 I42V<br>Spike: A67V Δ69–70 T95I G142D Δ143-145 NL211–212I 214EPE insertion G339D S371F S373P S375F K417N N440K G446S S477N T478K E484A Q493R G496R Q498R N501Y Y505H T547K D614G H655Y N679K P681H A701V D796Y N856K Q954H N969K L981F<br>Envelope: T9I<br>Membrane: D3G Q19E A63T<br>Nucleocapsid: P13L Δ31–33 RG203-204KR	-	Naturally occurring	[2]
SARS-CoV-2	Omicron variant, lineage BA.1, isolates:<br>hCoV-19/USA/NY-MSHSPSP-PV44488/2021<br>hCoV-19/USA/GA-EHC-2811C/2021<br>hCoV-19/Japan/NC928-2N/2021	NSP3: K38R SL1265–1266I A1892T<br>NSP4 T492I<br>NSP5 P132H<br>NSP6 Δ106–108<br>NSP12 P323L<br>NSP14 I42V<br>Spike: A67V Δ69-70 T95I G142D Δ143-145 NL211–212I 214EPE insertion G339D S371F S373P S375F K417N N440K G446S S477N T478K E484A Q493R G496R Q498R N501Y Y505H T547K D614G H655Y N679K	-	Naturally occurring	[2]

		P681H D796Y N856K Q954H N969K L981F Envelope: T9I Membrane: D3G Q19E A63T Nucleocapsid: P13L Δ31–33 RG203-204KR			
SARS-CoV-2	B.1.1.529/Omicron (R346K) (GenBank: OM212473) Lineage BA.1.1	NSP3: K38R SL1265-1266I A1892T NSP4 T492I NSP5 P132H NSP6: Δ105–107 I189V NSP12 P323L NSP14 I42V Spike: A67V Δ69–70 T95I G142D Δ143-145 NL211-212I 214EPE insertion G339 R346K S371L S373P S375F K417N N440K G446S S477N T478K E484A Q493R G496S Q498R N501Y Y505H T547K D614G H655Y N679K P681H N764K D796Y N856K Q954H N969K L981F Envelope T9I Membrane: D3G Q19E A63T Nucleocapsid: P13L Δ31-33 R203K G204R	-	Naturally occurring	[3]
SARS-CoV-2	B.1.1.529/Omicron (GenBank: OM212472) SARS-CoV-2/human/HKG/HKU-691/2021 Lineage BA.1	NSP3: K38R SL1265-1266I A1892T NSP4 T492I NSP5 P132H NSP6: Δ105-107 I189V NSP12 P323L NSP14 I42V Spike: A67V Δ69–70 T95I G142D Δ143-145 NL211-212I 214EPE insertion G339D S371L S373P S375F K417N N440K G446S S477N T478K E484A Q493R G496S Q498R N501Y Y505H T547K D614G H655Y N679K P681H N764K D796Y N856K Q954H N969K L981F	-	Naturally occurring	[3]

		Envelope T9I<br>Membrane: D3G Q19E A63T<br>Nucleocapsid: P13L Δ31–33 R203K G204R			
SARS-CoV-2	SARS-CoV2 GZ69	Relative to AP66:<br>NSP3: P74S S370L<br>S protein: K74N I95T<br>N protein: R203K G204R<br><br>Relative to WuhanHu-1:<br>NSP3: P74S S370L<br>NSP12 P323L<br>S protein D614G<br>N protein: R203K G204R	-	Naturally occurring	[4]
SARS-CoV-2 HK-13	Del-Mut-1	Spike Δ679–688	-	Serial passage in Vero E6 cells	[5,6]
SARS-CoV-2 HK-13	Ca-DelMut	Spike: P25L V367F del679–688<br>NSP3 A578V<br>Envelope F20S<br>ORF8: V62L L84S	-	Serial passage in Vero E6 cells	[6]
SARS-CoV-2 SH01	Sdel	Spike Δ683–689	-	Serial passage in Vero E6 cells	[7]
SARS-CoV-2 strain England/2/2020 (VE6-T)	ΔCS	Spike: Δ679–686 V687I	-	Serial passage in Vero E6 cells	[8]
SARS-CoV-2 USA-WA1/2020	rSARS-CoV-2/ORFΔ3a	ORF3a deletion	-	Reverse genetics	[9]
SARS-CoV-2 USA-WA1/2020	rSARS-CoV-2/ORFΔ6	ORF6 deletion	-	Reverse genetics	[9]
SARS-CoV-2 USA-WA1/2020	rSARS-CoV-2/ORFΔ7a	ORF7a deletion	-	Reverse genetics	[9]
SARS-CoV-2 USA-WA1/2020	rSARS-CoV-2/ORFΔ7b	ORF7b deletion	-	Reverse genetics	[9]

SARS-CoV-2 USA-WA1/2020	ΔPRRA	Spike Δ681–684	-	Reverse genetics	[10]
SARS-CoV-2 USA-WA1/2020	CDX-005/COVI-VAC	Codon pair deoptimized region: 24106–25378 (nt)<br>Spike Δ678–689	-	Reverse genetics	[11]
SARS-CoV-2 USA-WA1/2020 mNeonGreen (SARS-CoV-2 USA-WA1/2020 expressing mNeonGreen reporter)	ΔPRRA mNeonGreen	Spike Δ681–684	-	Reverse genetics	[10]
SARS-CoV-2 USA-WA1/2020 mNeonGreen (SARS-CoV-2 USA-WA1/2020 expressing mNeonGreen reporter)	ΔORF3-E virion	Envelope deletion<br>ORF3 deletion<br>TRS replacement (ACGAAC to CCGGAU)	-	Reverse genetics	[12]
SARS-CoV-2 USA-WA1/2020 mNeonGreen (SARS-CoV-2 USA-WA1/2020 expressing mNeonGreen reporter)	ΔORF3-E P10 virion	Envelope deletion<br>ORF3 deletion<br>TRS replacement (ACGAAC to CCGGAU)<br>NSP1 Y97N<br>NSP4 K35I<br>Spike P812R	-	Reverse genetics+ passaging in complementary cell line	[12]
SARS-CoV-2 USA-WA1/2020 mNeonGreen (SARS-CoV-2 USA-WA1/2020 expressing mNeonGreen reporter)	S-IV-P5-Vero-P2 virion	Envelope deletion<br>ORF3 deletion<br>TRS replacement (ACGAAC to CCGGAU)<br>NSP15 A54T<br>NSP16 D108N<br>Spike: N679D S689G N709D<br>Membrane: T130N	-	Reverse genetics+ passaging in complementary cell line and Vero E6 cells	[12]
SARS-CoV-2 WK-521	R685H	Spike R685H	-	Serial passage in Vero E6 cells	[13]
SARS-CoV-2 WK-521	del2	Spike Δ683–689	-	Serial passage in Vero E6 cells	[13]

SARS-CoV-2 Wuhan-Hu-1/2019	rSARS-CoV-2 nsp14 Y420A	NSP14 Y420A	-	Reverse genetics	[14]
SARS-CoV-2 Wuhan-Hu-1/2019	sCPD9	Codon pair deoptimized region: 20358–21503 (nt)	-	Reverse genetics	[15,16]
SARS-CoV-2 Wuhan-Hu-1/2019	sCPD10	Codon pair deoptimized region: 24308–25306 (nt)	-	Reverse genetics	[15]
SARS-CoV-2 Wuhan-Hu-1/2019	CPD6	Codon pair deoptimized regions: 11969–13450; 13953–14306 (nt)	-	Reverse genetics	[15]
SARS-CoV-2/human/Korea/CNUHV03/2020	CoV-2-CNUHV03-CA22°C	NSP2: Δ82–84 M85V G233E<br>NSP3: N1184D G1585D S1922N A2372V P2725L A2753V<br>NSP4: S2871G P2972S G2985R<br>NSP6: F3606L N3609K<br>NSP7 D3926A<br>NSP12: R4608W S4806N A5155V<br>NSP13: L5604F V5849I<br>NSP16: C7038S P7059S<br>Spike: C38Y S86F G96E G151S S968A<br>ORF7a: Y67C<br>Nucleocapsid: I361K	-	Serial passage in Vero E6 cells - cold-adaptation	[17]
SARS-CoV MA15	mutPBM/rSARS-CoV-MA15-(E-PBM-)	Envelope DLLV73–76GGGG	Envelope DLLV72–75GGGG	Reverse genetics	[18–20]
SARS-CoV MA15	N15A/rSARS-CoV-E-N15A	Envelope N15A	Envelope N15A	Reverse genetics	[18,21]
MERS-CoV EMC	rMERS-CoV-ΔE	Envelope protein deletion	Envelope protein deletion	Reverse genetics	[22]
MERS-MA30	rMERS-MA30-ΔE	Envelope protein deletion	Envelope protein deletion	Reverse genetics	[22]
MERS-MA30-Δ5 (MERS-MA30 with ORF5 deletion)	rMERS-MA30-Δ[5, E]	Envelope protein deletion	Envelope protein deletion	Reverse genetics	[22]

rMERS-MA30-Δ[3, 4a, 4b, 5] (MERS-MA30 with ORF3-5 deletion)	rMERS-MA30-Δ[3, 4a, 4b, 5, E]	Envelope protein deletion	Envelope protein deletion	Reverse genetics	[22]
rSARS-CoV-Urbani-Δ[6–9b] (SARS-CoV Urbani with ORF6–9b deletion)	rSARS-CoV-Δ[E, 6–9b]	Envelope protein deletion	Envelope protein deletion	Reverse genetics	[23]
SARS-CoV MA15	rMA15-ΔE/rSARS-CoV-ΔE/rSARS-CoV-MA15-ΔE	Envelope protein deletion	Envelope protein deletion	Reverse genetics	[18,20,21.24–26]
SARS-CoV Urbani	rSARS-CoV-ΔE (rU-ΔE)	Envelope protein deletion	Envelope protein deletion	Reverse genetics	[23,24,27–30]
SARS-CoV MA15	V25F/rSARS-CoV-EV25F	Envelope V25F	Envelope V25F	Reverse genetics	[18,21]
SARS-CoV MA15	Δ2	Envelope Δ38-45	Envelope Δ38-45	Reverse genetics	[18,26]
SARS-CoV MA15	Δ3	Envelope Δ46-52	Envelope Δ46-52	Reverse genetics	[18,26]
SARS-CoV MA15	Mut 1	Envelope: S3A V5L T9A T11A	Envelope: S3A V5L T9A T11A	Reverse genetics	[18,26]
MHV-GP[1]	MHV-Flt3L/gp	murine Flt3L gene inserted between Orf1ab and Spike genes	mouse/human Flt3L gene inserted between Orf1ab and Spike genes	Reverse genetics	[31]
MHV-GP[1]	MHV-GM/GP	murine GM-CSF gene inserted between Orf1ab and Spike genes	mouse/human GM-CSF gene inserted between Orf1ab and Spike genes	Reverse genetics	[32]
MHV-MelA[2]	MHV-GM/MelA	murine GM-CSF gene inserted between Orf1ab and Spike genes	mouse/human GM-CSF gene inserted between Orf1ab and Spike genes	Reverse genetics	[32]
MHV-GP[1]	MHV-IL15/gp	murine IL-15 gene inserted between Orf1ab and Spike genes	mouse/human IL-15 gene inserted between Orf1ab and Spike genes	Reverse genetics	[31]
MHV-GP[1]	MHV-IL2/gp	murine IL-2 gene inserted between Orf1ab and Spike genes	mouse/human IL-2 gene inserted between Orf1ab and Spike genes	Reverse genetics	[31]

MHV JHM.WU	rWu.nsp1 K194	NSP1 K194R	NSP1 K125R	Reverse genetics	[33]
MHV-A59	A59.nsp1 K194R	NSP1 K194R	NSP1 K125R	Reverse genetics	[33]
SARS-CoV MA15	rSARS-CoV-nsp1-ΔC	NSP1 Δ122-130	NSP1 Δ122-130	Reverse genetics	[18]
MHV JHM.WU	rWu.nsp1 R193S/K194E	NSP1: R193S K194E	NSP1: R124S K125E	Reverse genetics	[33]
MHV-A59	A59.nsp1 R193S/K194E	NSP1: R193S K194E	NSP1: R124S K125E	Reverse genetics	[33]
MHV-A59	F476L MHV	NSP12 F476L	NSP12 F480L	Reverse genetics (based on passaged remdesivir-resistant MHV virus)	[34]
MHV-A59	V553L MHV	NSP12 V553L	NSP12 V557L	Reverse genetics (based on passaged remdesivir-resistant MHV virus)	[34]
MHV-A59	F476L V553L MHV	NSP12: F476L V553L	NSP12: F480L V557L	Reverse genetics (based on passaged remdesivir-resistant MHV virus)	[34]
SARS-CoV MA15 NanoLuc (SARS-CoV MA15 expressing nanoluciferase with ORF7a/7b deletion)	F480L V557L SARS-CoV	NSP12: F480L V557L	NSP12: F480L V557L	Reverse genetics (based on passaged remdesivir-	[34]

				resistant MHV virus)	
MHV JHM.WU	rWU.nsp13 A335V	NSP13 A335V	NSP13 A336V	Reverse genetics	[33]
MHV-A59	D330A	NSP14 D330A	NSP14 D331A	Reverse genetics	[14,35]
MHV-A59	MHV nsp14-N380A	NSP14 N380A	NSP14 N386A	Reverse genetics	[14]
IBV M41R-nsp10.14rep	M41R-nsp10rep	NSP14 V393L	NSP14 V398L	Reverse genetics	[36]
MHV-A59	Y414A/rMHVnsp14-Y414A	NSP14 Y414A	NSP14 Y420A	Reverse genetics	[14,35]
MHV-A59	VUSS2	NSP14 Y414H	NSP14 Y420H	Reverse genetics	[37]
IBV strain YN	rSD-H223A	NSP15 H223A	NSP15 H234A	Reverse genetics	[38]
MHV-A59	N15m3	NSP15 H262A	NSP15 H234A	Reverse genetics	[39]
PEDV Colorado	icPEDV-EnUmt	NSP15 H226A	NSP15 H234A	Reverse genetics	[40]
HCoV-229E	HCoV-229E H250A	NSP15 H250A	NSP15 H249A	Reverse genetics	[41]
IBV strain YN	rSD-H238A	NSP15 H238A	NSP15 H249A	Reverse genetics	[38]
MHV-A59	MHV H277A	NSP15 H277A	NSP15 H249A	Reverse genetics	[41]
IBV strain YN	rSD-K278A	NSP15 K278A	NSP15 K289A	Reverse genetics	[38]
IBV strain YN	rSD-Y334A	NSP15 Y334A	NSP15 Y342A	Reverse genetics	[38]
IBV strain YN	rSD-H223A/H238A/K278A/Y334A	NSP15: H223A H238A K278A Y334A	NSP15: H234A H249A K289A Y342A	Reverse genetics	[38]

HCoV-229E	HCoV-D129A	NSP16 D129A	NSP16 D130A	Reverse genetics	[42]
MERS-CoV EMC	dNSP16	NSP16 D130A	NSP16 D130A	Reverse genetics	[43]
MERS-CoV MA1	dNSP16	NSP16 D130A	NSP16 D130A	Reverse genetics	[43]
MHV-A59	MHV-D130A	NSP16 D130A	NSP16 D130A	Reverse genetics	[42]
PEDV PC22A	D129A	NSP16 D129A	NSP16 D130A	Reverse genetics	[44]
SARS-CoV MA15	dNSP16	NSP16 D130A	NSP16 D130A	Reverse genetics	[45,46]
SARS-CoV Urbani	D130A	NSP16 D130A	NSP16 D130A	Reverse genetics	[45]
SARS-CoV Urbani	K170A	NSP16 K170A	NSP16 K170A	Reverse genetics	[45]
SARS-CoV Urbani	K46A	NSP16 K46A	NSP16 K46A	Reverse genetics	[45]
MHV-A59	MHV-Y15A	NSP16 Y15A	NSP16 Y15A	Reverse genetics	[42]
PEDV PC22A	KDKE4A	NSP16: K45A D129A K169A E202A	NSP16: K46A D130A K170A E203A	Reverse genetics	[44]
GFP-expressing MHV-JHM IA	D1329A	NSP3 D497A	NSP3 D226A	Reverse genetics	[47]
SARS-CoV MA15	D1022A	NSP3 D204A	NSP3 D226A	Reverse genetics	[48]
SARS-CoV MA15	G1130V	NSP3 G312V	NSP3 G334V	Reverse genetics	[48]
SARS-CoV MA15	H1045A	NSP3 H227A	NSP3 H249A	Reverse genetics	[48]
GFP-expressing MHV-JHM IA	N1347A/N1347A MHV	NSP3 N515A	NSP3 N244A	Reverse genetics	[47,49–51]

MHV-A59	MHV-N1348A	NSP3 N516A	NSP3 N244A	Reverse genetics	[52]
SARS-CoV MA15	N1040A	NSP3 N222A	NSP3 N244A	Reverse genetics	[48]
GFP-expressing MHV-JHM IA	A1438T/G1439V	NSP3: A606G G607V	NSP3: A333G G334V	Reverse genetics	[47]
SARS-CoV MA15	rSARS-CoV-MA15-Δ3a	ORF3a deletion	ORF3a deletion	Reverse genetics	[19]
rSARS-CoV-MA15-Δ3a (SARS-CoV MA15 with ORF3a deletion)	rSARS-CoV-MA15-(Δ3a,EΔ1)	ORF3a deletion<br>E protein: S3A V5L T9A T11A	ORF3a deletion<br>E protein: S3A V5L T9A T11A	Reverse genetics	[19]
SARS-CoV MA15	rSARS-CoV-MA15-((3a,E]-PBM-)	ORF3a SVPL271–274GMSM<br>E protein DLLV73–76GGGG	ORF3a SVPL272–275GMSM<br>E protein DLLV72–75GGGG	Reverse genetics	[19]
SARS-CoV MA15	rSARS-CoV-MA15-3a-TMD2-	ORF3a: S40A S58A	ORF3a: S40A S58A	Reverse genetics	[19]
SARS-CoV MA15	rSARS-CoV-MA15-3a-TMD[2,3]-	ORF3a: Y109A Y113A Q116A	ORF3a: Y109A Y113A Q116A	Reverse genetics	[19]
SARS-CoV MA15	rSARS-CoV-MA15-3a-TMD3-	ORF3a: Y91A H93A	ORF3a: Y91A H93A	Reverse genetics	[19]
SJHM-RA59/S4R29<br>MHV-A59 expressing MHV-JHM spike	SJHM-L1114R	Spike L1114R	Spike L1012R	Reverse genetics	[53]
SARS-CoV Urbani	T332I	Spike T332I	Spike T345I	Serial passage in Vero E6 cells - S109.8 antibody escape mutant	[54]
SJHM-RA59/S4R29<br>MHV-A59 expressing MHV-JHM spike	SJHM-HR1/R120/R121	Spike: Q1067H Q1094H L1114R	Spike: Q965H Q992H L1012R	Reverse genetics	[53,55]
SARS-CoV MA15	CRG3-MA	TRS replacement (ACGAAC to CCGGAU)	TRS replacement (ACGAAC to CCGGAU)	Reverse genetics	[56]

SARS-CoV Urbani	CRG3	TRS replacement (ACGAAC to CCGGAU)	TRS replacement (ACGAAC to CCGGAU)	Reverse genetics	[56]
SARS-CoV MA15	CRG7-MA	TRS replacement (ACGAAC to UGGUCGC)	TRS replacement (ACGAAC to UGGUCGC)	Reverse genetics	[56]

[1]MHV-A59-based vector lacking NS2, HE protein, ORF4, ORF5a and E protein and with 99-nt deletion in NSP1 and encoding a LCMV epitope gp33-41 and EGFP fusion protein; [2]MHV-A59-based vector lacking NS2, HE protein, ORF4, ORF5a and E protein and with 99-nt deletion in NSP1 and encoding a human melanoma Mel-A26-35 epitope and EGFP fusion protein

Table S2b. A summary of the results of *in vivo* experiments involving the included viruses excluding experiments involving knock-out animal models.

Parental (WT) virus	Mutations/virus name	Model	Dose, route if not intranasal	Infection outcome (compared to WT if presented)	Reference
SARS-CoV-2	strain TY38-873 (Omicron) Lineage BA.1	4-week-old Syrian hamsters	10^5 $TCID_{50}$	No weight loss, less severe histopathological changes in lungs, less severe pulmonary mechanics changes, no blood oxygen saturation drop, lower viral loads	[1]
SARS-CoV-2	Omicron variant, strain B.1.1.529, isolate hCoV-19/USA/WI-WSLH-221686/2021 Lineage BA.1	5-month-old female K18-hACE2 mice	10^5 FFU	No weight loss	[2]
		Syrian hamsters	10^3 PFU	Reduced viral load in lungs and nasal wash	
		K18-hACE2 Syrian hamsters	10^3 PFU	Reduced mortality and viral titers	
SARS-CoV-2	hCoV-19/USA/NY-MSHSPSP-PV44476/2021 Lineage BA.1	6–8-week-old female 129S1 mice	10^4 PFU	No weight loss, reduced viral titers	[2]
		6-month-old female K18-hACE2 mice	10^4 PFU	No weight loss, reduced lung CXCL10 and IL-6 concentrations, no increase in lung TNF-α and IFN-γ levels	
SARS-CoV-2	Omicron variant, lineage BA.1, isolates: hCoV-19/USA/NY-MSHSPSP-PV44488/2021 hCoV-19/USA/GA-EHC-2811C/2021 hCoV-19/Japan/NC928-2N/2021	6–8-week-old female 129S1 mice	10^4 PFU	No weight loss, reduced lung viral titer	[2]
		10–20-week-old male and female 129S1 mice	10^6 PFU	No weight loss, reduced lung viral titers	
		10–14-month-old C57BL/6 mice	10^5 PFU	No weight loss	
		6-week-old BALB/c mice	10^5 PFU	Reduced lung and nasal turbinate viral titers, no changes in pulmonary mechanics	
		Syrian hamsters	10^3 PFU	Reduced lung CT pathology, reduced lung viral titers, no	

				changes in lung respiratory mechanics	
		Syrian hamsters	10^3 PFU	No weight loss	
		Syrian hamsters	10^4 PFU	No weight loss, reduced viral load in nasal wash	
		Syrian hamsters	10^3 PFU	Lung inflammation but no other lung pathology	
SARS-CoV-2	B.1.1.529/Omicron (GenBank: OM212472) SARS-CoV-2/human/HKG/HKU-691/2021 Lineage BA.1	6–8-week-old male and female K18-hACE2 mice	2×10^3 PFU	Reduced lung and nasal viral loads and titers, reduced lung pathology, reduced lung CXCL10 and IFN-γ expression	[3]
		6–8-week-old male and female K18-hACE2 mice	2×10^3 PFU	Reduced mortality and weight loss	
		6–8-week-old female C57BL/6J mice	10^5 PFU	Reduced lung and nasal viral titers and loads	
SARS-CoV-2	SARS-CoV2 GZ69	Human patient	Unknown (Natural infection)	E gene Ct: 11,6; RdRP gene Ct: 13,9; N gene Ct: 15,5; no symptoms	[4]
SARS-CoV-2 HK-13	Spike Δ679-688	7–8-week-old golden Syrian hamsters	1.5×10^5 PFU	Reduced lung and tracheal viral titer, no weight loss, reduced lung pathology	[5]
SARS-CoV-2 HK-13	Spike: P25L V367F del679-688 NSP3 A578V Envelope F20S ORF8: V62L L84S	7–8-week-old golden Syrian hamsters	10^3 PFU	Decreased lung viral titers, no weight loss, no lung IFN-γ, TNF-α, IL-6 expression increase	[6]
SARS-CoV-2 SH01	Spike Δ683–689	7–8-week-old male golden Syrian hamsters	5×10^5 PFU	No weight loss, reduced viral titers in nasal turbinates, trachea and lungs, reduced viral loads in different tissues	[7]
SARS-CoV-2 strain England/2/2020 (VE6-T)	Spike: Δ679–686 V687I	16–20-week-old outbred female ferrets	10^5 PFU	Reduced viral titer and viral load in nasal washes	[8]

SARS-CoV-2 USA-WA1/2020	ORF3a deletion	6–8 week-old K18-hACE2 mice	10^5 PFU	Slightly reduced mortality, reduced lung viral titers, reduced lung pathology, decreased IFN-α and IFN-γ levels, lower IL-6/IL-10 ratio	[9]
SARS-CoV-2 USA-WA1/2020	ORF6 deletion	6–8 week-old K18-hACE2 mice	10^5 PFU	Reduced mortality, reduced lung pathology, decreased IFN-γ levels	[9]
SARS-CoV-2 USA-WA1/2020	ORF7a deletion	6–8 week-old K18-hACE2 mice	10^5 PFU	Reduced mortality, decreased IFN-γ levels	[9]
SARS-CoV-2 USA-WA1/2020	ORF7b deletion	6–8 week-old K18-hACE2 mice	10^5 PFU	Reduced mortality, decreased IFN-α levels, increased TNF-α concentrations at later time points	[9]
SARS-CoV-2 USA-WA1/2020	Spike Δ681–684	7–8 week-old male golden Syrian hamsters	10^5 PFU	No weight loss, no reduced activity	[10]
		5–9-week-old K18-hACE2 mice	10^3 PFU	Reduced weight loss, milder changes in pulmonary mechanics, reduced lung pathology, reduced lung IFN-γ and CXCL10 concentrations, increased lung IL-1β and IL-6 concentrations	
SARS-CoV-2 USA-WA1/2020	Codon pair deoptimized region: 24106–25378 (nt) Spike Δ678–689	5–6-week-old male golden Syrian hamster	5×10^4 PFU	No weight loss, no lung pathology other than inflammation	[11]
SARS-CoV-2 USA-WA1/2020 mNeonGreen (SARS-CoV-2 USA-WA1/2020 expressing mNeonGreen reporter)	Envelope deletion ORF3 deletion TRS replacement (ACGAAC to CCGGAU)	4–5-week-old golden Syrian hamsters	10^5 $TCID_{50}$	No weight loss, no detectable disease, reduced viral load	[12]
		7–9-week-old K18-hACE2 mice	3×10^5 $TCID_{50}$	No weight loss or death	
		7–9-week-old K18-hACE2 mice	6×10^4 $TCID_{50}$, intracranial	No morbidity, no mortality, no weight loss	

SARS-CoV-2 USA-WA1/2020 mNeonGreen (SARS-CoV-2 USA-WA1/2020 expressing mNeonGreen reporter)	Envelope deletion<br>ORF3 deletion<br>TRS replacement (ACGAAC to CCGGAU)<br>NSP15 A54T<br>NSP16 D108N<br>Spike: N679D S689G N709D<br>Membrane: T130N	4–5-week-old golden Syrian hamsters	5×10^3 $TCID_{50}$	No weight loss, no detectable disease	[12]
		7–9-week-old K18-hACE2 mice	2.5×10^3 $TCID_{50}$	No weight loss or death	
		7–9-week-old K18-hACE2 mice	500 $TCID_{50}$, intracranial	No morbidity, no mortality, no weight loss	
SARS-CoV-2 WK-521	Spike R685H	4–6-week-old male golden Syrian hamsters	1.5×10^4 PFU	No weight loss, reduced lung microscopic pathology and gross pathology, reduced expression of IFN-γ, IL-6 and CXCL10 in lungs, reduced viral titers in nasal turbinates and lungs	[13]
SARS-CoV-2 WK-521	Spike Δ683–689	4–6-week-old male golden Syrian hamsters	1.5×10^4 PFU	No weight loss, reduced lung microscopic pathology and gross pathology, reduced expression of IFN-γ, IL-6 and CXCL10 in lungs, reduced viral titers in nasal turbinates and lungs	[13]
SARS-CoV-2 Wuhan-Hu-1/2019	NSP14 Y420A	8-week-old K18-hACE2 mice	10^3 PFU	No mortality, no or reduced weight loss, reduced lung pathology,	[14]
SARS-CoV-2 Wuhan-Hu-1/2019	Codon pair deoptimized region: 20358–21503 (nt)	6-week-old golden Syrian hamsters	10^4 FFU	Reduced weight loss, no signs of infection, reduced lung pathology and inflammation	[15]
		5–7-week-old Roborovski dwarf hamsters	10^5 FFU	Minimal weight loss, no signs of infection	

SARS-CoV-2 Wuhan-Hu-1/2019	Codon pair deoptimized region: 24308–25306 (nt)	6-week-old golden Syrian hamsters	10^4 FFU	Reduced weight loss, no signs of infection	[15]
SARS-CoV-2 Wuhan-Hu-1/2019	Codon pair deoptimized regions: 11969–13450; 13953–14306 (nt)	6-week-old golden Syrian hamsters	10^5 FFU	Reduced lung pathology and inflammation, reduced weight loss	[15]
SARS-CoV-2/human/Korea/CNUHV03/2020	CoV-2-CNUHV03-CA22°C	5–6-week-old female K18-hACE2 mice	2×10^4 PFU	No mortality, reduced weight loss, reduced lung inflammation, reduced lung viral loads, no virus in extrapulmonary tissues	[17]
SARS-CoV MA15	Envelope DLLV73–76GGGG	16-week-old female BALB/c mice	10^5 PFU	No mortality, reduced weight loss, no lung gross pathology and minimal lung pathology and pneumonia, no increase in CXCL10 and IL-6 expression	[18–20]
SARS-CoV MA15	Envelope N15A	16-week-old female BALB/c mice	10^5 PFU	Reduced mortality, reduced lung pathology, smaller increase in airway IL-1β, TNF-α and IL-6 concentrations	[18,21]
MERS-CoV EMC	Envelope protein deletion	16–24-week-old K18-hDPP4 mice	5×10^3 FFU	No mortality, reduced weight loss	[22]
MERS-MA30	Envelope protein deletion	16–24-week-old hDPP4-KI mice	10^4 FFU	No mortality or weight loss	[22]
MERS-MA30-Δ5 (MERS-MA30 with ORF5 deletion)	Envelope protein deletion	16–24-week-old hDPP4-KI mice	10^4 FFU	No mortality or weight loss	[22]
rMERS-MA30-Δ[3, 4a, 4b, 5] (MERS-MA30 with ORF3-5 deletion)	Envelope protein deletion	16–24-week-old hDPP4-KI mice	10^4 FFU	Parental virus was already highly attenuated, no changes in virulence based on published data	[22]
		6–8-week-old BALB/c mice	1.2×10^4 PFU	No mortality or weight loss	[23,29]

rSARS-CoV-Urbani-Δ[6–9b] (SARS-CoV Urbani with ORF6–9b deletion)	Envelope protein deletion	hACE2-Tg mice	1.2×10^4 PFU	No mortality, no weight loss, no neuroinfection	
SARS-CoV MA15	Envelope protein deletion	6-week-old BALB/c mice	5×10^4 PFU	No mortality or weight loss	[18,20,21.24–26]
		12-month-old BALB/c mice	5×10^4 PFU	No mortality or weight loss	
		6-week-old BALB/c mice	6×10^3 PFU	No mortality, lung inflammation	
		12-month-old BALB/c mice	6×10^3 PFU	No mortality, no clinical disease, lung inflammation	
		18-month-old BALB/c mice	6×10^3 PFU	No mortality, no clinical disease, lung inflammation, minimal weight loss	
		8-week-old female BALB/c mice	6×10^3 PFU	Reduced lung inflammation	
		16-week-old female BALB/c mice	10^5 PFU	No mortality or weight loss, reduced lung viral titers, reduced TNF-α, CXCL10 and IL-6 expression, no disease symptoms, no lung gross pathology, reduced lung pathology and inflammation,	
		16-week-old BALB/c mice	10^5 PFU	No mortality, no weight loss, no signs of disease, reduced lung pathology and inflammation, smaller lung TNF, CSCL10 and IL-6 expression	
SARS-CoV Urbani	Envelope protein deletion	Golden Syrian hamsters	10^4 $TCID_{50}$	Reduced lung inflammation, no reduction in animal activity, reduced viral titers	[23,24,27–30]
		12-month-old BALB/c mice	6×10^3 PFU	No mortality or weight loss, limited lung pathology	
		6–8-week-old BALB/c mice	1.2×10^4 PFU	No mortality or weight loss	

		hACE2-Tg mice	$1.2{\times}10^4$ PFU	No mortality, no weight loss, no brain infection,	
SARS-CoV MA15	Envelope V25F	16-week-old female BALB/c mice	$1.2{\times}10^4$ PFU	Slightly reduced mortality	[21]
SARS-CoV MA15	Envelope Δ38–45	16-week-old BALB/c mice	10^5 PFU	No mortality, no weight loss, no signs of disease, reduced lung viral titers, reduced lung damage and inflammation	[26]
SARS-CoV MA15	Envelope Δ46–52	16-week-old BALB/c mice	10^5 PFU	No mortality, no weight loss, no signs of disease, reduced lung viral titers, reduced lung damage and inflammation, reduced lung TNF, CXCL10, IL-6 and IFN-γ concentrations	[26]
SARS-CoV MA15	Envelope: S3A V5L T9A T11A	16-week-old BALB/c mice	10^5 PFU	No mortality, reduced weight loss, no signs of disease, reduced lung damage and inflammation	[26]
MHV-GP[1]	murine Flt3L gene inserted between Orf1ab and Spike genes	6–9 week-old C57BL/6 mice	10^6 PFU	Not described, parental virus is already highly attenuated	[31]
MHV-GP[1]	murine GM-CSF gene inserted between Orf1ab and Spike genes	6–9 week-old C57BL/6 mice	10^6 PFU	Not described, parental virus is already highly attenuated	[31]
MHV-MelA[2]	murine GM-CSF gene inserted between Orf1ab and Spike genes	6–9 week-old C57BL/6 mice	10^6 PFU	Not described, parental virus is already highly attenuated	[32]
MHV-GP[1]	murine IL-15 gene inserted between Orf1ab and Spike genes	6–9 week-old C57BL/6 mice	10^6 PFU	Not described, parental virus is already highly attenuated	[32]
MHV-GP[1]	murine IL-2 gene inserted between Orf1ab and Spike genes	6–9 week-old C57BL/6 mice	10^6 PFU	Not described, parental virus is already highly attenuated	[31]

MHV JHM.WU	NSP1 K194R	4-week-old C57BL/6 mice	500 PFU intrahepatic	Reduced liver viral titer	[33]
MHV-A59	NSP1 K194R	4-week-old C57BL/6 mice	5000 PFU intrahepatic	Reduced liver viral titer	[33]
SARS-CoV MA15	NSP1 Δ122-130	16-week-old female BALB/c mice	10^5 PFU	No mortality, reduced weight loss, no gross lung pathology, no lung damage	[18]
MHV-A59	NSP1: R193S K194E	4-week-old C57BL/6 mice	5000 PFU intrahepatic	Reduced liver viral titer	[33]
SARS-CoV MA15 NanoLuc (SARS-CoV MA15 expressing nanoluciferase with ORF7a/7b deletion)	NSP12: F480L V557L	10-week-old female BALB/c mice	10^3 PFU	No significant difference	[34]
		10-week-old female BALB/c mice	10^4 PFU	Reduced lung viral titer, reduced weight loss	
MHV JHM.WU	NSP13 A335V	4-week-old C57BL/6 mice	500 PFU intrahepatic	Reduced liver viral titer	[33]
MHV-A59	NSP14 D330A	4-week-old male C57BL/6 mice	2×10^6 PFU intrahepatic	No mortality, no weight loss, minimal liver inflammation and no liver fibrosis, increased serum IFN-β levels at 12 hpi but not 24 hpi,	[14,35]
		4-week-old male C57BL/6 mice	5×10^5 PFU subcutaneous	No significant difference – parental virus did not produce disease	
IBV M41R-nsp10.14rep	NSP14 V393L	8-days old chickens	10^5 PFU intraocular+intranasal	Shorter duration of infection, reduced symptoms, smaller reduction in ciliary activity	[36]
MHV-A59	NSP14 Y414A	4-week-old male C57BL/6 mice	2×10^6 PFU intrahepatic	No mortality, no weight loss, minimal liver inflammation and no liver fibrosis, increased serum IFN-β levels at 12 hpi but not 24 hpi,	[14,35]
		4-week-old male C57BL/6 mice	5×10^5 PFU subcutaneous	No significant difference – parental virus did not produce disease	
		4-week-old male C57BL/6 mice	10^4 PFU intrahepatic	No mortality, minor increase in serum ALT, no	

				neuroinfection, no liver pathology, increased serum IFN-β concentrations at 1 dpi but lower at 3 dpi	
		4-week-old male C57BL/6 mice	10^4 PFU intracranial	No mortality, minor increase in serum ALT, no liver infection, no liver pathology, smaller increase in serum IFN-β concentrations	
		4-week-old male C57BL/6 mice	7.5×10^5 PFU intrahepatic	Earlier alteration in gene expression in liver	
MHV-A59	NSP14 Y414H	4-week-old C57BL/6 mice	Intracranial	Increased LD_{50}	[37]
		4-week-old C57BL/6 mice	5×10^4 PFU intracranial	Reduced brain viral titers, no liver infection	
MHV-A59	NSP15 H262A	6-week-old female C57BL/6 mice	10^4 PFU intraperitoneal	No mortality, no viral titer in liver or spleen, reduced weight loss, no liver pathology	[39]
PEDV Colorado	NSP15 H226A	7-day-old piglets	10^5 $TCID_{50}$ oral	reduced viral shedding, no mortality, similar disease and pathology	[40]
IBV strain YN	NSP15 H238A	1-day-old chickens	10^6 EID_{50} intraocular	No clinical signs of disease, reduced ciliary injury, reduced viral loads, no tracheal, lung and kidney pathology	[38]
MHV-A59	NSP15 H277A	8–10-week-old C57BL/6 mice	500 PFU intraperitoneal	No virus in spleen or liver	[41]
IBV strain YN	NSP15 Y334A	1-day-old chickens	10^6 EID_{50} intraocular	No clinical signs of disease, reduced ciliary injury, reduced viral loads, no tracheal, lung and kidney pathology	[38]

MERS-CoV EMC	NSP16 D130A	Ad5-hDPP4-transduced 10-to-20-week-old BALB/c mice	10^6 PFU	No viral replication at 2 dpi, parental virus was already non-pathogenic	[43]
		10–20-week-old Dpp4 288–330$^{+/+}$ mice	10^6 PFU	Reduced IFN-β, IFN-α4 and IFN-α7 expression, parental virus was already non-pathogenic	
MERS-CoV MA1	NSP16 D130A	10–20-week-old Dpp4 288–330$^{+/+}$ mice	10^6 PFU	No mortality, reduced weight loss, reduced lung pathology	[43]
MHV-A59	NSP16 D130A	6–9 week-old C57BL/6 mice	500 PFU intraperitoneal	No virus present in spleen and liver at 2 dpi	[42]
SARS-CoV MA15	NSP16 D130A	10-week-old female BALB/c mice	10^5 PFU	No mortality, no significant pulmonary mechanics changes,	
		10-week-old C57BL/6 mice	10^5 PFU	Reduced weight loss, reduced lung pathology	
		10-week-old female BALB/c mice	100 PFU	Minimal weight loss	
		20 week-old C57BL/6 mice	No data	No mortality, disease course similar until 4 dpi	
		12-month-old female BALB/c mice	10^5 PFU	Lethal infection, but viral titer reduced at 4 dpi	
		12-month-old female BALB/c mice	100 PFU	Reduced weight loss, no mortality, reduced lung viral titer, reduced changes in pulmonary mechanics	
MHV-A59	NSP16 Y15A	6–9 week-old C57BL/6 mice	500 PFU intraperitoneal	No virus present in spleen and liver at 2 dpi	[42]
PEDV PC22A	NSP16: K45A D129A K169A E202A	4-days-old piglets	100 PFU oral	Reduced mortality, reduced intestinal pathology, lower and delayed peak titer of shed virus	[44]

GFP-expressing MHV-JHM IA	NSP3 D497A	5–8-week-old C57BL/6 mice	3×10^3 PFU	Reduced mortality	[47]
SARS-CoV MA15	NSP3 D204A	7–9 week-old female BALB/c mice	3×10^4 PFU	Reduced mortality and weight loss	[48]
SARS-CoV MA15	NSP3 G312V	7–9 week-old female BALB/c mice	3×10^4 PFU	No mortality, reduced weight loss	[48]
SARS-CoV MA15	NSP3 H227A	7–9 week-old female BALB/c mice	3×10^4 PFU	Reduced mortality and weight loss	[48]
GFP-expressing MHV-JHM IA	NSP3 N515A	5–8-week-old C57BL/6 mice	3×10^4 PFU	No mortality, no weight loss	[47.50]
		5–8-week-old male C57BL/6 mice	4×10^4 PFU	No mortality, no weight loss, reduced brain viral titers, reduced expression of IFN-β, IL-6 and TNF-α in brains, reduced brain pathology	
		5–8-week-old male C57BL/6 mice	750 PFU	No mortality, reduced weight loss	
MHV-A59	NSP3 N516A	6–9-week-old C57BL/6 mice	$5–5\times10^5$ PFU intraperitoneal	No liver pathology, reduced serum ALT increase	[52]
		6–9-week-old 129Sv mice	5 PFU intraperitoneal	Decreased liver and spleen IFN-α levels, decreased liver and spleen IL-6	
SARS-CoV MA15	NSP3 N222A	7–9 week-old female BALB/c mice	3×10^4 PFU	Reduced mortality, reduced weight loss, larger increase in IFN-α, IFN-β, CXCL-10, IL-6 and TNF expression in lungs at 24 hpi but not at 72 hpi, reduced lung pathology	[48]
GFP-expressing MHV-JHM IA	NSP3: A606G G607V	5–8-week-old male C57BL/6 mice	3×10^3 PFU	No mortality, no weight loss	[47]
SARS-CoV MA15	ORF3a deletion	16-week-old BALB/c female mice	10^5 PFU	No mortality, reduced weight loss, reduced lung viral titer, reduced disease symptoms	[19]

[1]MHV-A59-based vector lacking NS2, HE protein, ORF4, ORF5a and E protein and with 99-nt deletion in NSP1 and encoding a LCMV epitope gp33-41 and EGFP fusion protein; [2]MHV-A59-based vector lacking NS2, HE protein, ORF4, ORF5a and E protein and with 99-nt deletion in NSP1

SARS-CoV MA15	ORF3a SVPL271–274GMSM<br>E protein DLLV73–76GGGG	16-week-old BALB/c female mice	10^5 PFU	No mortality, reduced weight loss	[19]
SARS-CoV MA15	ORF3a: S40A S58A	16-week-old BALB/c female mice	10^5 PFU	Slightly reduced mortality	[19]
SARS-CoV MA15	ORF3a: Y109A Y113A Q116A	16-week-old BALB/c female mice	10^5 PFU	No mortality, reduced weight loss	[19]
SARS-CoV MA15	ORF3a: Y91A H93A	16-week-old BALB/c female mice	10^5 PFU	No mortality, reduced weight loss, reduced lung viral titer	[19]
SJHM-RA59/S4R29<br>MHV-A59 expressing MHV-JHM spike	Spike L1114R	4-week-old male C57BL/6 mice	Varied, intranasal	Increased dose required to cause symptomatic disease, reduced replication and inflammation in brain, replication restricted to olfactory bulb	[53]
		4-week-old male C57BL/6 mice	Varied, intracranial	Increased dose required to cause symptomatic disease, reduced replication and inflammation in brain, replication restricted to olfactory bulb	
SARS-CoV Urbani	Spike T332I	12-month-old male BALB/c mice	10^5 PFU	No mortality, reduced weight loss, no lung pathology other than inflammation	[54]
SJHM-RA59/S4R29<br>MHV-A59 expressing MHV-JHM spike	Spike: Q1067H Q1094H L1114R	4-week-old male C57BL/6 mice	Varied, intranasal	Increased dose required to cause symptomatic disease, increased LD_{50}, reduced replication and inflammation in brain, replication restricted to olfactory bulb	[53,55]
		4-week-old male C57BL/6 mice	Varied, intracranial	Increased dose required to cause symptomatic disease,	

				increased LD_{50}, reduced replication and inflammation in brain, replication restricted to olfactory bulb,	
		4-week-old male C57BL/6 mice	10 PFU intracranial	Reduced brain viral titers	
		4-week-old male C57BL/6 mice	5×10^4 PFU intranasal	Reduced brain viral titer at 3 dpi but increased at 5 dpi	
SARS-CoV MA15	TRS replacement (ACGAAC to CCGGAU)	10-week-old female BALB/c	Not specified	Reduced mortality	[56]
		12-month-old female BALB/c mice	Not specified	Reduced mortality	
SARS-CoV Urbani	TRS replacement (ACGAAC to CCGGAU)	10-week-old female BALB/c	Not specified	No significant difference	[56]
		12-month-old female BALB/c mice	Not specified	No weight loss	
SARS-CoV MA15	TRS replacement (ACGAAC to UGGUCGC)	10-week-old female BALB/c	Not specified	No mortality, reduced lung viral titer	[56]
		12-month-old female BALB/c mice	10^2 PFU	No mortality	
		12-month-old female BALB/c mice	10^3 PFU	No mortality	
		12-month-old female BALB/c mice	10^4 PFU	Reduced mortality	

and encoding a human melanoma Mel-A26-35 epitope and EGFP fusion protein. Data on IFN, TNF, IL-1 and IL-6 cytokines as well as CXCL10 chemokine was selected for presentation.

Table S2c. A summary of the results of experiments that aimed to study the reversion to virulence of attenuated coronaviruses *in vitro*.

Virus	Mutations	Corresponding mutations in SARS-CoV-2	Conditions	Results	Reference
SARS-CoV Urbani	ΔEnvelope	ΔEnvelope	16 passages in Vero E6 cells	Spike S607F on passage 5. Minimal lethality after 16 passages.	[29]
SARS-CoV Urbani with with deletion of ORF3-9b	ΔEnvelope	ΔEnvelope	16 passages in Vero E6 cells	Spike S607F and NSP8 T89I were detected.	[29]
MERS-MA30 with deletion of ORF3, 4a, 4b and 5	ΔEnvelope	ΔEnvelope	5 passages in complementary cell line expressing E protein	No evidence of recombination with cellular E protein RNA	[22]
SARS-CoV-2	Codon pair deoptimized region: 20358–21503	NA	10 passages in Vero E6 cells	No phenotypic change or mutations in codon pair deoptimized sequences	[15]
SARS-CoV-2	Codon pair deoptimized region: 24308–25306	NA	10 passages in Vero E6 cells	No phenotypic change or mutations in codon pair deoptimized sequences	[15]
MHV-A59	NSP3 N516A	NSP3 N244A	12 passages in L929 cells	No reversion	[52]
SARS-CoV MA15	ΔEnvelope	ΔEnvelope	16 passages in Vero E6 cells	Virus gained a new protein with PDM-binding motif. Cell-line specific titer increase but no change in virulence.	[18]
SARS-CoV MA15	ΔEnvelope	ΔEnvelope	16 passages in DBT-mACE2	Virus gained a new protein with PDM-binding motif. Cell-line specific titer increase but no change in virulence.	[18]
SARS-CoV MA15	Envelope Δ38–45	Envelope Δ38–45	8 or 16 passages in Vero E6 cells	No significant changes	[18,26]
SARS-CoV MA15	Envelope Δ46–52	Envelope Δ46–52	8 or 16 passages in Vero E6 cells	No significant changes	[18,26]
SARS-CoV MA15	Envelope: S3A, V5L, T9A, T11A	Envelope: S3A, V5L, T9A, T11A	8 or 16 passages in Vero E6 cells	No significant changes	[18,26]

SARS-CoV MA15	Envelope N15A	Envelope N15A	24 passages in Vero E6 cells	Reversion through A15D mutation at passage 24	[21]
MHV-A59	NSP14 D330A	NSP14 D331A	10 passages in Neuro 2a cells	No reversion	[35]
MHV-A59	NSP14 Y414A	NSP14 Y420A	10 passages in Neuro 2a cells	No reversion	[35]
SARS-CoV MA15	Envelope V25F	Envelope V25F	24 passages in Vero E6	Incorporation of reverting mutations at passage 8	[21]
SARS-CoV MA15	Envelope DLLV73–76GGGG	Envelope DLLV72–75GGGG	16 passages in Vero E6 cells	The virus gained a novel PDZ-binding motif in E protein	[18]
MHV-A59	NSP14 N380A	NSP14 N386A	Cell culture	Reversion after 1 passage	[14]
SARS-CoV-2	Envelope deletion<br>ORF3 deletion<br>TRS replacement<br>(ACGAAC to CCGGAU)	-	10 passages in complementary cell line expressing E and ORF3a proteins	ΔORF3-E P10 virion replicon was obtained with NSP1 Y97N, NSP4 K35I and Spike P812R mutations. The resultant replicon propagated to a higher infectious titer in complementary cell line.	[12]
SARS-CoV-2	Envelope deletion<br>ORF3 deletion<br>TRS replacement<br>(ACGAAC to CCGGAU)	-	5 passages in complementary cell line expressing E and ORF3a proteins, 2 passages in Vero E6 cells	S-IV-P5-Vero-P2 virion with NSP15 A54T, NSP16 D108N, Spike: N679D S689G N709D and Membrane: T130N mutations was obtained. It could be propagated in non-complementary Vero E6 cell line.	[12]
SARS-CoV-2	Envelope deletion<br>ORF3 deletion<br>TRS replacement<br>(ACGAAC to CCGGAU)<br>NSP15 A54T<br>NSP16 D108N<br>Spike: N679D S689G N709D<br>Membrane: T130N	-	10 passages in Vero E6 cells	No improved replication	[12]

Table S2d. A summary of the results of experiments that aimed to study immunogenicity of attenuated coronaviruses.

Virus	Mutations	Corresponding mutations in SARS-CoV-2	Animal, route of administration, dose	Endpoint	Result (attenuated/WT)	Reference
MHV-MelA	murine GM-CSF gene insertion	Murine/human GM-CSF gene insertion	A2DR1 mice, intravenous, 10^5 PFU	Tet-MelA+ CD8+ T cells/spleen at 28 dpi	1,07×10^5/ND	[32]
SARS-CoV MA15	NSP16 D130A	NSP16 D130A	BALB/c mice, Intranasal, 10^5 PFU	SARS-CoV MA15 serum 50% neutralizing titer	>1600/ND;	[45,46]
				SHC014-MA15 serum 50% neutralizing titer	>800/ND	
SARS-CoV Urbani	TRS replacement (ACGAAC to CCGGAU)	TRS replacement (ACGAAC to CCGGAU)	10-week-old BALB/c mice, Intranasal, 10^2-10^3 PFU	SARS-CoV Urbani serum 50% neutralizing titer	4820/ND	[56]
SARS-CoV Urbani	TRS replacement (ACGAAC to CCGGAU)	TRS replacement (ACGAAC to CCGGAU)	12-month-old BALB/c mice, Intranasal, 10^2-10^3 PFU	SARS-CoV Urbani serum 50% neutralizing titer	6420/ND	[56]
SARS-CoV MA15	ΔEnvelope	ΔEnvelope	6-week-old BALB/c mice, Intranasal, 6×10^3 PFU	SARS-CoV MA15 serum 50% neutralizing titer at 21 and 66 dpi	91 and 648/ND	[24]
				S366 peptide specific lung CD8+ IFN-γ+ cells at 7 dpi	0,8%/ND	
SARS-CoV MA15	ΔEnvelope	ΔEnvelope	12-month-old BALB/c mice, Intranasal, 6×10^3 PFU	SARS-CoV MA15 serum 50% neutralizing titer at 21 and 66 dpi	198 and 1260/ND	[24]
				S366 peptide specific lung CD8+ IFN-γ+ cells at 7 dpi	3,7%/ND	
MHV-A59	NSP14 D330A	NSP14 D331A	4-week-old C57BL/6 mice, subcutaneous, 5×10^5PFU	MHV-A59 serum 50% neutralizing titer at 28 and 336 dpi	244 and 67/618 and 251	[35]
				Virus specific IFN-γ+ CD8+ splenocytes at 28 dpi	0,79%/0,82%	

MHV-A59	NSP14 D330A	NSP14 D331A	4-week-old C57BL/6 mice, subcutaneous, 5×10^5 PFU	MHV-A59 serum 50% neutralizing titer at 28 and 336 dpi	432 and 126/618 and 251	[35]
				Virus specific IFN-γ+ CD8+ splenocytes at 28 dpi	0,95%/0,82%	
MERS-CoV EMC	NSP16 D130A	NSP16 D130A	10–20-week-old Dpp4 288–330$^{+/+}$ mice, intranasal, 10^6 PFU	MERS-CoV EMC serum 50%neutralizing titer	>800/ND	[43]
MHV-GP	murine Flt3L gene insertion	Murine/human Flt3L gene insertion	C57BL/6 mice, intravenous, 10^5 PFU	Tet-gp34+ CD8+ splenocytes at 7 dpi	10,32%/2,69%	[31]
MHV-GP	murine GM-CSF gene insertion	Murine/human GM-CSF gene insertion	C57BL/6 mice, intravenous, 10^5 PFU	Tet-gp34+ CD8+ splenocytes at 7 dpi	2,79%/1.20%	[32]
MHV-GP	murine IL-15 gene insertion	Murine/human IL-15 gene insertion	C57BL/6 mice, intravenous, 10^5 PFU	Tet-gp34+ CD8+ splenocytes at 7 dpi	5,42%/2,69%	[31]
MHV-GP	murine IL-2 gene insertion	Murine/human IL-2 gene insertion	C57BL/6 mice, intravenous, 10^5 PFU	Tet-gp34+ CD8+ splenocytes at 7 dpi	5,33%/2,69%	[31]
MHV-MelA	murine GM-CSF gene insertion	Murine/human GM-CSF gene insertion	A2DR1 mice, intravenous, 10^5 PFU	Tet-MelA+ CD8+ T cells/spleen at 28 dpi	$1,07\times10^5$/ND	[32]
SARS-CoV-2	Cold adaptation	NA	K18-hACE2 mice, intranasal, 2×10^4 PFU	CoV-2-CNUHV03 serum 50% neutralizing titer at 19 dpi	640–4960/ND	[17]
				CoV-2-KCDC03 50% serum neutralizing titer at 19 dpi	640–4960/ND	
PEDV PC22A	NSP16: K45A D129A K169A E202A	NSP16: K46A, D130A, K170A, E202A	4-days old piglets, oral, 100 PFU	PEDV PC22A serum 50% neutralizing titer at 21 dpi	125/ND	[44]

SARS-CoV-2	Codon pair deoptimized region: 24106 - 25378 (nt) Spike Δ678-689	NA	5–6-week-old Syrian hamsters, intranasal, $5×10^3$ PFU	SARS-CoV-2 serum 50% neutralizing titer at 21 dpi	1260/1000	[11]
SARS-CoV-2	R685H	NA	5–6-week-old Syrian hamsters, intranasal, $1.5×10^4$ PFU	SARS-CoV-2 serum 50% neutralizing titer at 19 dpi	766/766	[13]
				Lineage P.1 serum 50% neutralizing titer at 19 dpi	274/229	
				Lineage B.1.1.7 serum 50% neutralizing titer at 19 dpi	2550/1560	
SARS-CoV-2	Spike Δ683–689	NA	5–6-week-old Syrian hamsters, intranasal, $1.5×10^4$ PFU	SARS-CoV-2 serum 50% neutralizing titer at 19 dpi	1360/766	[13]
				Lineage P.1 serum 50% neutralizing titer at 19 dpi	267/229	
				Lineage B.1.1.7 serum 50% neutralizing titer at 19 dpi	1710/1560	
SARS-CoV-2	Spike Δ681-684	NA	Syrian hamsters, intranasal, $5×10^5$ PFU	SARS-CoV-2 serum 50% neutralizing titer	637/575	[10]
SARS-CoV-2	Vero E6 passaged	NA	Syrian hamsters, intranasal, $1.25×10^5$ PFU	SARS-CoV-2 serum 50% neutralizing titer	243/211	[6]
SARS-CoV-2	Vero E6 passaged	NA	Ad5-hACE2 transduced 6–8 week-old BALB/c mice; intranasal; 10^5 PFU	Spike-specific IFN-γ+ CD8+ cells in lungs at 28 dpi	0,75%/ND	[6]
SARS-CoV Urbani	ΔEnvelope	ΔEnvelope	Syrian hamster, intranasal, 10^3 $TCID_{50}$	SARS-CoV Urbani serum 50% neutralizing titer	280/367	[27]
				SARS-CoV GD03 serum 50% neutralizing titer	52/45	
SARS-CoV Urbani	ΔEnvelope	ΔEnvelope	6-week-old BALB/c mice,	SARS-CoV MA15 serum 50% neutralizing titer at 21 and 66 dpi	<10/ND and <LOD/ND	[24]

			Intranasal, 6×10^3 PFU	S366 peptide specific lung CD8+ IFN-γ+ cells at 7 dpi	Very few/ND	
SARS-CoV Urbani	ΔEnvelope	ΔEnvelope	12-month-old BALB/c mice, Intranasal, 6×10^3 PFU	SARS-CoV MA15 serum 50% neutralizing titer at 21 and 66 dpi	<LOD/ND and <LOD/ND	[24]
				S366 peptide specific lung CD8+ IFN-γ+ cells at 7 dpi	0,03%/ND	
SARS-CoV Urbani	ΔEnvelope	ΔEnvelope	12-month-old BALB/c mice, Intranasal, 1.2×10^4 PFU	SARS-CoV Urbani serum 50% neutralizing titer at 21 dpi	≤10/ND	[29]
				S366 peptide specific blood CD8+ IFN-γ+ cells at 7 dpi	1,6%/ND	
SARS-CoV Urbani	ΔEnvelope	ΔEnvelope	hACE2-Tg mice, Intranasal; 1.2×10^4 PFU	SARS-CoV Urbani serum 50% neutralizing titer at 21 dpi	≤20/ND	[29]
				S366 peptide specific blood CD8+ IFN-γ+ cells at 7 dpi	2,37%/ND	
SARS-CoV Urbani with with deletion of ORF3-9b	ΔEnvelope	ΔEnvelope	12-month-old BALB/c mice, Intranasal, 1.2×10^4 PFU	SARS-CoV Urbani serum 50% neutralizing titer at 21 dpi	≤10/ND	[29]
				S366 peptide specific blood CD8+ IFN-γ+ cells at 7 dpi	1,7%/ND	
SARS-CoV Urbani with with deletion of ORF3-9b	ΔEnvelope	ΔEnvelope	hACE2-Tg mice, Intranasal; 1.2×10^4 PFU	SARS-CoV Urbani serum 50% neutralizing titer at 21 dpi	≤20/ND	[29]
				S366 peptide specific blood CD8+ IFN-γ+ cells at 7 dpi	1,3%/ND	
MERS-MA30 with deletion of ORF3, 4a, 4b and 5	ΔEnvelope	ΔEnvelope	16–24-week-old hDPP4-KI mice, Intranasal, 10^4 FFU	MERS-CoV MA30 serum 50% neutralizing titer	56,1/ND	[22]
SARS-CoV-2	Spike: Δ679–686, V687I	NA	16–20-week-old ferrets, intranasal, 10^5 PFU	SARS-CoV-2 serum 50% neutralizing titer	40-80/10-80	[8]

Table S2e. A summary of the results of the challenge studies.

Parental (WT) virus	Mutations/virus name	Model	Vaccine virus dose, vaccination route (if not intranasal)	Vaccination-challenge interval (days)	Challenge virus, dose, route	Difference in infection outcome	References
SARS-CoV-2 HK-13	Ca-DelMut	7–8-week-old golden Syrian hamsters	1.25×10^5 PFU	28	SARS-CoV-2 HK-13 and HK-95, 10^3 PFU	Highly reduced weight loss, highly reduced lung pathology	[6]
SARS-CoV-2 WK-521	Spike Δ683–689	4–6-week-old male golden Syrian hamsters	1.5×10^4 PFU	19	SARS-CoV-2 WK-521, 1.5×10^4 PFU	No weight loss, no macroscopic lung pathology, reduced viral load in lungs and nasal turbinates, no virus in nasal turbinates and lungs at 5 dpi, increased IFN-γ expression in lungs and decreased IL-6 expression in lungs	[13]
		4–6-week-old male golden Syrian hamsters	1.5×10^4 PFU	19	SARS-CoV-2 TY7-501 (lineage P.1), 1.5×10^4 PFU	No weight loss, no macroscopic lung pathology, reduced viral load in lungs and nasal turbinates, low viral titers in nasal turbinates at 5 dpi, virus not present in lungs at 5 dpi, decreased IL-6 expression in lungs	
		4–6-week-old male golden Syrian hamsters	1.5×10^4 PFU	19	SARS-CoV-2 QK002 (lineage B.1.1.7), 1.5×10^4 PFU	No weight loss, no macroscopic lung pathology, reduced viral load in lungs and nasal turbinates, low viral titers in nasal turbinates at 5 dpi, no virus in lungs at 5 dpi,	
SARS-CoV-2 WK-521	Spike R685H	4–6-week-old male golden Syrian hamsters	1.5×10^4 PFU	19	SARS-CoV-2 WK-521, 1.5×10^4 PFU	No weight loss, no macroscopic lung pathology, reduced viral load in lungs and nasal turbinates, no virus in nasal turbinates and lungs at 5 dpi, increased IFN-γ expression in lungs and decreased IL-6 expression in lungs	[13]
SARS-CoV-2 USA-WA1/2020	Codon pair deoptimized region: 24106–	5–6-week-old male golden Syrian hamsters	5×10^4 PFU	16	SARS-CoV-2 USA-WA1/2020, 5×10^4 PFU	Reduced lung viral titer, reduced viral titer in olfactory bulb, no neuroinfection, no significant weight loss	[11]

	25378 (nt) Spike Δ678-689						
SARS-CoV-2 Wuhan-Hu-1/2019	NSP14 Y420A	8-week-old K18-hACE2 mice	10^4 PFU	Unspecified	SARS-CoV-2 Wuhan-Hu-1/2019, 5×10^4 PFU	No mortality, no significant weight loss	[14]
SARS-CoV-2/human/ Korea/CNUHV03/2020	CoV-2-CNUHV03-CA22°C	5–6-week-old female K18-hAC2 mice	2×10^3 PFU	21	SARS-CoV-2- KCDC03, 2×10^4 PFU	No apparent weight loss, mild pneumonia, no detectable virus at 6 dpi	[17]
			2×10^4 PFU	21	SARS-CoV-2- KCDC03, 2×10^4 PFU	No apparent weight loss, no pneumonia, no detectable virus at 6 dpi	
SARS-CoV-2 USA-WA1/2020	Spike Δ681-684	7–8 week-old male golden Syrian hamsters	10^5 PFU	28	SARS-CoV-2 USA-WA1/2020, 10^5 PFU	No weight loss, no signs of disease, no virus detectable in nasal wash at 4 dpi	[10]
SARS-CoV-2 Wuhan-Hu-1/2019	Codon pair deoptimized region: 24308–25306 (nt)	6-week-old golden Syrian hamsters	10^4 FFU	21	SARS-CoV-2 lineage B.1, 10^5 FFU	No weight loss, no signs of disease, reduced lung pathology and inflammation, reduced viral loads, no live virus present in lungs at 2-5 dpi	[15]
SARS-CoV-2 Wuhan-Hu-1/2019	Codon pair deoptimized region: 20358–21503 (nt)	6-week-old golden Syrian hamsters	10^4 FFU	21	SARS-CoV-2 lineage B.1, 10^5 FFU	No weight loss, no signs of disease, reduced lung pathology and inflammation, reduced viral loads, no live virus present in lungs at 2-5 dpi	[15,16]
		5–7-week-old Roborovski dwarf hamsters	10^5 FFU	21	BetaCoV/Munich/ BavPat1/2020 (B.1 lineage), 10^5 PFU	No weight loss, no signs of disease, no lung pathology and inflammation, reduced viral loads, no live virus present in lungs at 2-5 dpi	
					BetaCoV/Germany/ ChVir21652/2020 (B.1.1.7 lineage), 10^5 PFU	No weight loss, no signs of disease, no lung pathology and inflammation, reduced viral loads, no live virus present in lungs at 2-5 dpi	
					hCoV-19/Netherlands/ NoordHolland_20159/ 2021 (B.1.351 lineage), 10^5 PFU	No weight loss, no signs of disease, no lung pathology and inflammation, reduced viral loads, no live virus present in lungs at 2-5 dpi in most animals	

SARS-CoV-2 Wuhan-Hu-1/2019	Codon pair deoptimized regions: 11969–13450; 13953–14306 (nt)	6 week-old golden Syrian hamsters	10^5 FFU	21	SARS-CoV-2 lineage B.1, 10^5 FFU	No weight loss, no signs of disease, reduced lung pathology and inflammation, reduced viral loads, no live virus present in lungs at 2-5 dpi	[15]
MERS-MA30-Δ5 (MERS-MA30 with ORF5 deletion)	Envelope protein deletion	16–24-week-old hDPP4-KI mice	10^4 FFU	21	MERS-MA30, 10^5 FFU	No mortality, no weight loss, reduced viral loads, no live virus in lungs at 2-6 dpi	[22]
rMERS-MA30-Δ[3, 4a, 4b, 5] (MERS-MA30 with ORF3-5 deletion)	Envelope protein deletion	16–24-week-old hDPP4-KI mice	10^4 FFU	21	MERS-MA30, 10^5 FFU	No mortality, no significant weight loss	[22]
MERS-MA30	Envelope protein deletion	16–24-week-old hDPP4-KI mice	10^4 FFU	21	MERS-MA30, 10^5 FFU	No mortality, no significant weight loss	[22]
MERS-CoV EMC	Envelope protein deletion	16–24-week-old K18-hDPP4 mice	5×10^3 FFU	21	MERS-CoV EMC, 5×10^4 FFU	No weight loss, no mortality	[22]
SARS-CoV Urbani	Envelope protein deletion	Golden Syrian hamsters	10^3 $TCID_{50}$	28	SARS-CoV Urbani, 10^3 $TCID_{50}$	Reduced lung inflammation, smaller reduction in animal activity, no live virus in lungs at 2 and 5 dpi, no live virus in nasal turbinates at 5 dpi	[24,27–30]
		6-week-old BALB/c mice	6×10^3 PFU	21	SARS-CoV MA15, 10^5 PFU	No mortality, reduced weight loss,	
		12-month-old BALB/c mice	6×10^3 PFU	21	SARS-CoV MA15, 10^5 PFU	Reduced mortality	
		6-week-old BALB/c mice	6×10^3 PFU	66	SARS-CoV MA15, 10^5 PFU	No difference	
		12-month-old BALB/c mice	6×10^3 PFU	66	SARS-CoV MA15, 10^5 PFU	No difference except small decrease in lung viral titer	
		6–8-week-old BALB/c mice	1.2×10^4 PFU	21	SARS-CoV MA15, 10^5 PFU	No mortality, reduced weight loss, reduced lung pathology	
		hACE2-Tg mice	1.2×10^4 PFU	21	SARS-CoV Urbani, 1.2×10^4 PFU	Reduced mortality	

References:

rSARS-CoV-Urbani-Δ[6–9b] (SARS-CoV Urbani with ORF6–9b deletion)	Envelope protein deletion	6–8-week-old BALB/c mice	1.2×10^4 PFU	21	SARS-CoV MA15, 10^5 PFU	No mortality, reduced weight loss, reduced lung pathology	[29]
		hACE2-Tg mice	1.2×10^4 PFU	21	SARS-CoV Urbani, 1.2×10^4 PFU	Reduced mortality	
SARS-CoV MA15	Envelope protein deletion	6-week-old BALB/c mice	6×10^3 PFU	21	SARS-CoV MA15, 10^5 PFU	No mortality, no weight loss, reduced vural titer, no detectable virus at 4 dpi	[24]
		12-month-old BALB/c mice	6×10^3 PFU	21	SARS-CoV MA15, 10^5 PFU	No mortality, no weight loss, reduced vural titer, no detectable virus at 4 dpi, highly reduced lung pathology and inflammation	
		18-month-old BALB/c mice	6×10^3 PFU	21	SARS-CoV MA15, 10^5 PFU	No mortality or weight loss	
		6-week-old BALB/c mice	6×10^3 PFU	66	SARS-CoV MA15, 10^5 PFU	No weight loss, reduced lung viral titer, no detectable virus at 4 dpi, reduced lung inflammation and pathology	
		12-month-old BALB/c mice	6×10^3 PFU	66	SARS-CoV MA15, 10^5 PFU	No weight loss, reduced lung viral titer, no detectable virus at 4 dpi, reduced lung inflammation and pathology	
SARS-CoV MA15	Envelope Δ38–45	6-week-old BALB/c mice	6×10^3 PFU	21	SARS-CoV MA15, 10^5 PFU	No mortality, no weight loss	[26]
SARS-CoV MA15	Envelope Δ46–52	6-week-old BALB/c mice	6×10^3 PFU	21	SARS-CoV MA15, 10^5 PFU	No mortality, no weight loss	[26]
SARS-CoV MA15	Envelope: S3A, V5L, T9A, T11A	6-week-old BALB/c mice	6×10^3 PFU	21	SARS-CoV MA15, 10^5 PFU	No mortality, no weight loss	[26]
SARS-CoV MA15	NSP1 Δ122–130	8-week-old female BALB/c mice	6×10^3 PFU	21	SARS-CoV MA15, 10^5 PFU	No mortality, no weight loss	[18]
MHV-A59	NSP14 D330A	4-week-old male C57BL/6 mice	5×10^5 PFU subcutaneous	30	MHV-A59, 2×10^7 PFU, intrahepatic	No mortality, no weight loss, no liver pathology, smaller serum ALT increase	[29]

MHV-A59	NSP14 Y414A	4-week-old male C57BL/6 mice	5×10^5 PFU subcutaneous	30	MHV-A59, 2×10^7 PFU, intrahepatic	No mortality, no weight loss, no liver pathology, smaller serum ALT increase	[29]
MHV-A59	NSP15 H262A	6-week-old C57BL/6 mice	6×10^4 PFU intraperitoneal	30	MHV-A59, 6×10^4 PFU, intraperitoneal	No liver pathology, no detectable virus in liver or spleen	[39]
MERS-CoV EMC	NSP16 D130A	10–20-week-old Dpp4 288–330$^{+/+}$ mice	10^6 PFU	28	MERS-CoV MA1, 10^6 PFU	No mortality, reduced weight loss, reduced lung viral titers, no lung pathology in most animals	[43]
SARS-CoV MA15	NSP16 D130A	10-week-old BALB/c mice	10^5 PFU	28	SARS-CoV MA15, 10^5 PFU	No mortality, reduced weight loss	[45,46]
		10-week-old BALB/c mice	10^5 PFU	28	SARS-CoV MA15 expressing SHC014-CoV-derived spike gene, 10^5 PFU	No weight loss, no lung pathology, no live virus in lungs at 4 dpi	
		12-month-old BALB/c mice	100 PFU	28	SARS-CoV MA15, 10^5 PFU	No mortality, no viral replication	
PEDV PC22A	NSP16: K45A D129A K169A E202A	4-days-old piglets	100 PFU, oral	21	PEDV icPC22A, 10^6 PFU, oral	No mortality, most animals were protected from symptoms	[44]
SARS-CoV Urbani	TRS replacement (ACGAAC to CCGGAU)	10-week-old female BALB/c	10^2–10^3 PFU	22	SARS-CoV MA15, 10^6 PFU	No weight loss, virus not detectable in lungs at 4 dpi	[56]
		10-week-old female BALB/c	10^2–10^3 PFU	22	SARS-CoV expressing HCSZ6103 spike, 10^6 PFU	Virus not detectable in lungs at 4 dpi	
		12-month-old female BALB/c mice	10^2–10^3 PFU	22	SARS-CoV MA15, 10^6 PFU	No weight loss, virus not detectable in lungs at 4 dpi	
		12-month-old female BALB/c mice	10^2–10^3 PFU	22	SARS-CoV expressing HCSZ6103 spike, 10^6 PFU	No weight loss, virus not detectable in lungs at 4 dpi	
SARS-CoV MA15	TRS replacement (ACGAAC to UGGUCGC)	12-month-old female BALB/c mice	$3{,}16\times10^2$ PFU	22	SARS-CoV MA15, 10^5 PFU	No mortality, no significant weight loss	[56]

1. Suzuki, R.; Yamasoba, D.; Kimura, I.; Wang, L.; Kishimoto, M.; Ito, J.; Morioka, Y.; Nao, N.; Nasser, H.; Uriu, K.; et al. Attenuated fusogenicity and pathogenicity of SARS-CoV-2 Omicron variant. *Nature* **2022**, *603*, 700–705. https://doi.org/10.1038/s41586-022-04462-1.
2. Halfmann, P. J.; Iida, S.; Iwatsuki-Horimoto, K.; Maemura, T.; Kiso, M.; Scheaffer, S. M.; Darling, T. L.; Joshi, A.; Loeber, S.; Singh, G.; et al. SARS-CoV-2 Omicron virus causes attenuated disease in mice and hamsters. *Nature* **2022**, 603, 687–692. https://doi.org/10.1038/s41586-022-04441-6.
3. Shuai, H.; Chan, J. F.-W.; Hu, B.; Chai, Y.; Yuen, T. T.-T.; Yin, F.; Huang, X.; Yoon, C.; Hu, J.-C.; Liu, H.; et al. Attenuated replication and pathogenicity of SARS-CoV-2 B.1.1.529 Omicron. *Nature* **2022**, *603*, 693–699. https://doi.org/10.1038/s41586-022-04442-5.
4. Caccuri, F.; Zani, A.; Messali, S.; Giovanetti, M.; Bugatti, A.; Campisi, G.; Filippini, F.; Scaltriti, E.; Ciccozzi, M.; Fiorentini, S.; et al. A persistently replicating SARS-CoV-2 variant derived from an asymptomatic individual. *Journal of Translational Medicine* **2020**, *18*, 362. https://doi.org/10.1186/s12967-020-02535-1.
5. Lau, S.-Y.; Wang, P.; Mok, B. W.-Y.; Zhang, A. J.; Chu, H.; Lee, A. C.-Y.; Deng, S.; Chen, P.; Chan, K.-H.; Song, W.; et al. Attenuated SARS-CoV-2 variants with deletions at the S1/S2 junction. *Emerging Microbes & Infections* **2020**, *9*, 837–842. https://doi.org/10.1080/22221751.2020.1756700.
6. Wang, P.; Lau, S.-Y.; Deng, S.; Chen, P.; Mok, B. W.-Y.; Zhang, A. J.; Lee, A. C.-Y.; Chan, K.-H.; Tam, R. C.-Y.; Xu, H.; et al. Characterization of an attenuated SARS-CoV-2 variant with a deletion at the S1/S2 junction of the spike protein. *Nature Communications* **2021**, *12*, 2790.
7. Zhu, Y.; Feng, F.; Hu, G.; Wang, Y.; Yu, Y.; Zhu, Y.; Xu, W.; Cai, X.; Sun, Z.; Han, W.; et al. A genome-wide CRISPR screen identifies host factors that regulate SARS-CoV-2 entry. *Nature Communications* **2021**, *12*, 961. https://doi.org/10.1038/s41467-021-21213-4.
8. Peacock, T. P.; Goldhill, D. H.; Zhou, J.; Baillon, L.; Frise, R.; Swann, O. C.; Kugathasan, R.; Penn, R.; Brown, J. C.; Sanchez-David, R. Y.; et al. The furin cleavage site in the SARS-CoV-2 spike protein is required for transmission in ferrets. *Nature Microbiology* **2021**, *6*, 899–909. https://doi.org/10.1038/s41564-021-00908-w.
9. Silvas, J. A.; Vasquez, D. M.; Park, J.-G.; Chiem, K.; Allué-Guardia, A.; Garcia-Vilanova, A.; Platt, R. N.; Miorin, L.; Kehrer, T.; Cupic, A.; et al. Contribution of SARS-CoV-2 Accessory Proteins to Viral Pathogenicity in K18 Human ACE2 Transgenic Mice. *Journal of Virology* **2021**, *95*, e00402-21. https://doi.org/10.1128/JVI.00402-21.
10. Johnson, B. A.; Xie, X.; Bailey, A. L.; Kalveram, B.; Lokugamage, K. G.; Muruato, A.; Zou, J.; Zhang, X.; Juelich, T.; Smith, J. K.; et al. Loss of furin cleavage site attenuates SARS-CoV-2 pathogenesis. *Nature* **2021**, *591*, 293–299. https://doi.org/10.1038/s41586-021-03237-4.
11. Wang, Y.; Yang, C.; Song, Y.; Coleman, J. R.; Stawowczyk, M.; Tafrova, J.; Tasker, S.; Boltz, D.; Baker, R.; Garcia, L.; et al. Scalable live-attenuated SARS-CoV-2 vaccine candidate demonstrates preclinical safety and efficacy. *Proc. Natl. Acad. Sci. U.S.A.* **2021**, *118*, e2102775118. https://doi.org/10.1073/pnas.2102775118.
12. Zhang, X.; Liu, Y.; Liu, J.; Bailey, A. L.; Plante, K. S.; Plante, J. A.; Zou, J.; Xia, H.; Bopp, N. E.; Aguilar, P. v.; et al. A trans-complementation system for SARS-CoV-2 recapitulates authentic viral replication without virulence. *Cell* **2021**, *184*, 2229-2238.e13. https://doi.org/10.1016/j.cell.2021.02.044.
13. Sasaki, M.; Toba, S.; Itakura, Y.; Chambaro, H. M.; Kishimoto, M.; Tabata, K.; Intaruck, K.; Uemura, K.; Sanaki, T.; Sato, A.; et al. SARS-CoV-2 Bearing a Mutation at the S1/S2 Cleavage Site Exhibits Attenuated Virulence and Confers Protective Immunity. *mBio* **2021**, *12*, e01415–21. https://doi.org/10.1128/mBio.01415-21.
14. Pan, R.; Kindler, E.; Cao, L.; Zhou, Y.; Zhang, Z.; Liu, Q.; Ebert, N.; Züst, R.; Sun, Y.; Gorbalenya, A.E.; et al. N7-Methylation of the Coronavirus RNA Cap Is Required for Maximal Virulence by Preventing Innate Immune Recognition. *mBio* **2022**, *13*, e0366221. https://doi.org/10.1128/mbio.03662-21.
15. Trimpert, J.; Dietert, K.; Firsching, T. C.; Ebert, N.; Thi Nhu Thao, T.; Vladimirova, D.; Kaufer, S.; Labroussaa, F.; Abdelgawad, A.; Conradie, A.; et al. Development of safe and highly protective live-attenuated SARS-CoV-2 vaccine candidates by genome recoding. *Cell Reports* **2021**, *36*, 109493. https://doi.org/10.1016/j.celrep.2021.109493.
16. Trimpert, J.; Adler, J. M.; Eschke, K.; Abdelgawad, A.; Firsching, T. C.; Ebert, N.; Thao, T. T. N.; Gruber, A. D.; Thiel, V.; Osterrieder, N.; et al. Live attenuated virus vaccine protects against SARS-CoV-2 variants of concern B.1.1.7 (Alpha) and B.1.351 (Beta). *Science Advances* **2021**, *7*, eabk0172. https://doi.org/10.1126/sciadv.abk0172.
17. Seo, S.; Jang, Y. Cold-Adapted Live Attenuated SARS-Cov-2 Vaccine Completely Protects Human ACE2 Transgenic Mice from SARS-Cov-2 Infection. *Vaccines* **2020**, *8*, 584. https://doi.org/10.3390/vaccines8040584.
18. Jimenez-Guardeño, J. M.; Regla-Nava, J. A.; Nieto-Torres, J. L.; DeDiego, M. L.; Castaño-Rodriguez, C.; Fernandez-Delgado, R.; Perlman, S.; Enjuanes, L. Identification of the Mechanisms Causing Reversion to Virulence in an Attenuated SARS-CoV for the Design of a Genetically Stable Vaccine. *PLoS Pathogens* **2015**, *11*, e1005215. https://doi.org/10.1371/journal.ppat.1005215.

19. Castaño-Rodriguez, C.; Honrubia, J.M.; Gutiérrez-Álvarez, J.; DeDiego, M.L.; Nieto-Torres, J.L.; Jimenez-Guardeño, J.M.; Regla-Nava, J.A.; Fernandez-Delgado, R.; Verdia-Báguena, C.; Queralt-Martín, M.; et al. Role of Severe Acute Respiratory Syndrome Coronavirus Viroporins E, 3a, and 8a in Replication and Pathogenesis. *mBio* **2018**, *9*. https://doi.org/10.1128/mbio.02325-17.
20. Jimenez-Guardeño, J.M.; Nieto-Torres, J.L.; DeDiego, M.L.; Regla-Nava, J.A.; Fernandez-Delgado, R.; Castaño-Rodriguez, C.; Enjuanes, L. The PDZ-Binding Motif of Severe Acute Respiratory Syndrome Coronavirus Envelope Protein Is a Determinant of Viral Pathogenesis. *PLOS Pathog.* **2014**, *10*, e1004320. https://doi.org/10.1371/journal.ppat.1004320.
21. Nieto-Torres, J. L.; DeDiego, M. L.; Verdiá-Báguena, C.; Jimenez-Guardeño, J. M.; Regla-Nava, J. A.; Fernandez-Delgado, R.; Castaño-Rodriguez, C.; Alcaraz, A.; Torres, J.; Aguilella, V. M.; et al. Severe Acute Respiratory Syndrome Coronavirus Envelope Protein Ion Channel Activity Promotes Virus Fitness and Pathogenesis. *PLoS Pathogens* **2014**, *10*, e1004077. https://doi.org/10.1371/journal.ppat.1004077.
22. Gutiérrez-Álvarez, J.; Honrubia, J.M.; Sanz-Bravo, A.; González-Miranda, E.; Fernández-Delgado, R.; Rejas, M.T.; Zúñiga, S.; Sola, I.; Enjuanes, L. Middle East respiratory syndrome coronavirus vaccine based on a propagation-defective RNA replicon elicited sterilizing immunity in mice. *Proc. Natl. Acad. Sci. U.S.A.* **2021**, *118*. https://doi.org/10.1073/pnas.2111075118.
23. DeDiego, M. L.; Pewe, L.; Alvarez, E.; Rejas, M. T.; Perlman, S.; Enjuanes, L. Pathogenicity of severe acute respiratory coronavirus deletion mutants in hACE-2 transgenic mice. *Virology* **2008**, *376*. 379-389. https://doi.org/10.1016/j.virol.2008.03.005.
24. Fett, C.; DeDiego, M.L.; Regla-Nava, J.A.; Enjuanes, L.; Perlman, S. Complete Protection against Severe Acute Respiratory Syndrome Coronavirus-Mediated Lethal Respiratory Disease in Aged Mice by Immunization with a Mouse-Adapted Virus Lacking E Protein. *J. Virol.* **2013**, *87*, 6551–6559. https://doi.org/10.1128/jvi.00087-13.
25. DeDiego, M.L.; Nieto-Torres, J.L.; Regla-Nava, J.A.; Jimenez-Guardeño, J.M.; Fernandez-Delgado, R.; Fett, C.; Castaño-Rodriguez, C.; Perlman, S.; Enjuanes, L. Inhibition of NF-κB-Mediated Inflammation in Severe Acute Respiratory Syndrome Coronavirus-Infected Mice Increases Survival. *J. Virol.* **2014**, *88*, 913–924. https://doi.org/10.1128/jvi.02576-13.
26. Regla-Nava, J.A.; Nieto-Torres, J.L.; Jimenez-Guardeño, J.M.; Fernandez-Delgado, R.; Fett, C.; Castaño-Rodríguez, C.; Perlman, S.; Enjuanes, L.; DeDiego, M.L. Severe Acute Respiratory Syndrome Coronaviruses with Mutations in the E Protein Are Attenuated and Promising Vaccine Candidates. *J. Virol.* **2015**, *89*, 3870–3887. https://doi.org/10.1128/jvi.03566-14.
27. Lamirande, E.W.; DeDiego, M.L.; Roberts, A.; Jackson, J.P.; Alvarez, E.; Sheahan, T.; Shieh, W.-J.; Zaki, S.R.; Baric, R.; Enjuanes, L.; et al. A Live Attenuated Severe Acute Respiratory Syndrome Coronavirus Is Immunogenic and Efficacious in Golden Syrian Hamsters. *J. Virol.* **2008**, *82*, 7721–7724. https://doi.org/10.1128/jvi.00304-08.
28. DeDiego, M.L.; Álvarez, E.; Almazán, F.; Rejas, M.T.; Lamirande, E.; Roberts, A.; Shieh, W.-J.; Zaki, S.R.; Subbarao, K.; Enjuanes, L. A Severe Acute Respiratory Syndrome Coronavirus That Lacks the E Gene Is Attenuated In Vitro and In Vivo. *J. Virol.* **2007**, *81*, 1701–1713. https://doi.org/10.1128/jvi.01467-06.
29. Netland, J.; DeDiego, M.L.; Zhao, J.; Fett, C.; Álvarez, E.; Nieto-Torres, J.L.; Enjuanes, L.; Perlman, S. Immunization with an attenuated severe acute respiratory syndrome coronavirus deleted in E protein protects against lethal respiratory disease. *Virology* **2010**, *399*, 120–128. https://doi.org/10.1016/j.virol.2010.01.004.
30. Enjuanes, L.; Nieto-Torres, J.L.; Jimenez-Guardeño, J.M.; DeDiego, M.L. Recombinant Live Vaccines to Protect Against the Severe Acute Respiratory Syndrome Coronavirus. **2010**, 73–97. https://doi.org/10.1007/978-3-0346-0277-8_4.
31. Perez-Shibayama, C.; Gil-Cruz, C.; Nussbacher, M.; Allgäuer, E.; Cervantes-Barragan, L.; Züst, R.; Ludewig, B. Dendritic Cell-Specific Delivery of Flt3L by Coronavirus Vectors Secures Induction of Therapeutic Antitumor Immunity. *PLoS ONE* **2013**, *8*, e81442. https://doi.org/10.1371/journal.pone.0081442.
32. Cervantes-Barragan, L.; Züst, R.; Maier, R.; Sierro, S.; Janda, J.; Levy, F.; Speiser, D.; Romero, P.; Rohrlich, P. S.; Ludewig, B.; et al. Dendritic cell-specific antigen delivery by coronavirus vaccine vectors induces long-lasting protective antiviral and antitumor immunity. *mBio* **2010**, *1*, e00171-10. https://doi.org/10.1128/mBio.00171-10.
33. Zhang, R.; Li, Y.; Cowley, T.J.; Steinbrenner, A.; Phillips, J.M.; Yount, B.L.; Baric, R.S.; Weiss, S.R. The nsp1, nsp13, and M Proteins Contribute to the Hepatotropism of Murine Coronavirus JHM.WU. *J. Virol.* **2015**, *89*, 3598–3609. https://doi.org/10.1128/jvi.03535-14.
34. Agostini, M.L.; Andres, E.L.; Sims, A.C.; Graham, R.L.; Sheahan, T.P.; Lu, X.; Smith, E.C.; Case, J.B.; Feng, J.Y.; Jordan, R.; et al. Coronavirus Susceptibility to the Antiviral Remdesivir (GS-5734) Is Mediated by the Viral Polymerase and the Proofreading Exoribonuclease. *mBio* **2018**, *9*, e00221-18. https://doi.org/10.1128/mbio.00221-18.

35. Zhang, Z.; Liu, Q.; Sun, Y.; Li, J.; Liu, J.; Pan, R.; Cao, L.; Chen, X.; Li, Y.; Xu, K.; et al. Live attenuated coronavirus vaccines deficient in N7-Methyltransferase activity induce both humoral and cellular immune responses in mice. *Emerg. Microbes Infect.* **2021**, *10*, 1626–1637. https://doi.org/10.1080/22221751.2021.1964385.
36. Keep, S.; Stevenson-Leggett, P.; Dowgier, G.; Everest, H.; Freimanis, G.; Oade, M.; Hammond, J.A.; Armesto, M.; Vila, R.; Bru, T.; et al. Identification of amino acids within non-structural proteins 10 and 14 of the avian coronavirus infectious bronchitis virus that result in attenuation *in vivo* and *in ovo*. *J. Virol.* **2022**, *96*, e0205921. https://doi.org/10.1128/jvi.02059-21.
37. Sperry, S.M.; Kazi, L.; Graham, R.L.; Baric, R.S.; Weiss, S.R.; Denison, M.R. Single-Amino-Acid Substitutions in Open Reading Frame (ORF) 1b-nsp14 and ORF 2a Proteins of the Coronavirus Mouse Hepatitis Virus Are Attenuating in Mice. *J. Virol.* **2005**, *79*, 3391–3400. https://doi.org/10.1128/jvi.79.6.3391-3400.2005.
38. Jing, Z.; Lu, S.; Ye, Z.; Delan, F.; Jinlong, C.; Guozhong, Z.; Kanta, S. Coronavirus Endoribonuclease Ensures Efficient Viral Replication and Prevents Protein Kinase R Activation. *Journal of Virology* **2021**, *95*, e02103–20.
39. Deng, X.; Hackbart, M.; Mettelman, R.C.; O'Brien, A.; Mielech, A.M.; Yi, G.; Kao, C.C.; Baker, S.C. Coronavirus nonstructural protein 15 mediates evasion of dsRNA sensors and limits apoptosis in macrophages. *Proc. Natl. Acad. Sci. U.S.A.* **2017**, *114*, E4251–E4260. https://doi.org/10.1073/pnas.1618310114.
40. Xufang, D.; Albert, van G.; C., B. A.; Amornrat, O.; Angela, P.; M., L. K.; S., F. K.; C., B. S.; Susana, L. Coronavirus Endoribonuclease Activity in Porcine Epidemic Diarrhea Virus Suppresses Type I and Type III Interferon Responses. *J. Virol* **2019**, *93*, e02000–18. https://doi.org/10.1128/JVI.02000-18.
41. Kindler, E.; Gil Cruz, C.; Spanier, J.; Li, Y.; Wilhelm, J.; Rabouw, H.H.; Züst, R.; Hwang, M.; V'Kovski, P.; Stalder, H.; et al. Early endonuclease-mediated evasion of RNA sensing ensures efficient coronavirus replication. *PLOS Pathog.* **2017**, *13*, e1006195. https://doi.org/10.1371/journal.ppat.1006195.
42. Zust, R.; Cervantes-Barragan, L.; Habjan, M.; Maier, R.; Neuman, B.W.; Ziebuhr, J.; Szretter, K.J.; Baker, S.C.; Barchet, W.; Diamond, M.S.; et al. Ribose 2′-O-methylation provides a molecular signature for the distinction of self and non-self mRNA dependent on the RNA sensor Mda5. *Nat. Immunol.* **2011**, *12*, 137–143. https://doi.org/10.1038/ni.1979.
43. Menachery, V.D.; Gralinski, L.E.; Mitchell, H.D.; Dinnon, K.H., III; Leist, S.R.; Yount, B.L., Jr.; Graham, R.L.; McAnarney, E.T.; Stratton, K.G.; Cockrell, A.S.; et al. Middle East Respiratory Syndrome Coronavirus Nonstructural Protein 16 Is Necessary for Interferon Resistance and Viral Pathogenesis. *mSphere* **2017**, *2*, e00346-17. https://doi.org/10.1128/msphere.00346-17.
44. Yixuan, H.; Hanzhong, K.; Jineui, K.; Dongwan, Y.; Yunfang, S.; Patricia, B.; Juliet, C.; N., V. A.; J., S. L.; Qiuhong, W.; et al. Engineering a Live Attenuated Porcine Epidemic Diarrhea Virus Vaccine Candidate via Inactivation of the Viral 2′-O-Methyltransferase and the Endocytosis Signal of the Spike Protein. *Journal of Virology* **2019**, *93*, e00406–19.
45. Menachery, V. D.; Yount, B. L.; Josset, L.; Gralinski, L. E.; Scobey, T.; Agnihothram, S.; Katze, M. G.; Baric, R. S. Attenuation and Restoration of Severe Acute Respiratory Syndrome Coronavirus Mutant Lacking 2′-O-Methyltransferase Activity. *Journal of Virology* **2014**, *88*, 4251–4264. https://doi.org/10.1128/JVI.03571-13.
46. Menachery, V.D.; Gralinski, L.E.; Mitchell, H.D.; Dinnon, K.H., III; Leist, S.R.; Yount, B.L., Jr.; McAnarney, E.T.; Graham, R.L.; Waters, K.M.; Baric, R.S. Combination Attenuation Offers Strategy for Live Attenuated Coronavirus Vaccines. *J. Virol.* **2018**, *92*. https://doi.org/10.1128/jvi.00710-18.
47. Voth, L. S.; O'Connor, J. J.; Kerr, C. M.; Doerger, E.; Schwarting, N.; Sperstad, P.; Johnson, D. K.; Fehr, A. R. Unique Mutations in the Murine Hepatitis Virus Macrodomain Differentially Attenuate Virus Replication, Indicating Multiple Roles for the Macrodomain in Coronavirus Replication. *Journal of Virology* **2021**, *95*, 766–787. https://doi.org/10.1128/jvi.00766-21.
48. Fehr, A.R.; Channappanavar, R.; Jankevicius, G.; Fett, C.; Zhao, J.; Athmer, J.; Meyerholz, D.K.; Ahel, I.; Perlman, S. The Conserved Coronavirus Macrodomain Promotes Virulence and Suppresses the Innate Immune Response during Severe Acute Respiratory Syndrome Coronavirus Infection. *mBio* **2016**, *7*, e01721-16. https://doi.org/10.1128/mbio.01721-16.
49. Grunewald, M.E.; Chen, Y.; Kuny, C.; Maejima, T.; Lease, R.; Ferraris, D.; Aikawa, M.; Sullivan, C.S.; Perlman, S.; Fehr, A.R. The coronavirus macrodomain is required to prevent PARP-mediated inhibition of virus replication and enhancement of IFN expression. *PLOS Pathog.* **2019**, *15*, e1007756. https://doi.org/10.1371/journal.ppat.1007756.
50. Fehr, A.R.; Athmer, J.; Channappanavar, R.; Phillips, J.M.; Meyerholz, D.K.; Perlman, S. The nsp3 Macrodomain Promotes Virulence in Mice with Coronavirus-Induced Encephalitis. *J. Virol.* **2014**, *89*, 1523–1536. https://doi.org/10.1128/jvi.02596-14.

51. Wheeler, D. L.; Sariol, A.; Meyerholz, D. K.; Perlman, S. Microglia are required for protection against lethal coronavirus encephalitis in mice. *Journal of Clinical Investigation* **2018**, *128*, 931-943, https://doi.org/10.1172/JCI97229.
52. Eriksson, K. K.; Cervantes-Barragán, L.; Ludewig, B.; Thiel, V. Mouse Hepatitis Virus Liver Pathology Is Dependent on ADP-Ribose-1″-Phosphatase, a Viral Function Conserved in the Alpha-Like Supergroup. *Journal of Virology* **2008**, *82*, 12325–12334.
53. Tsai, J.C.; de Groot, L.; Pinon, J.D.; Iacono, K.T.; Phillips, J.J.; Seo, S.-H.; Lavi, E.; Weiss, S.R. Amino acid substitutions within the heptad repeat domain 1 of murine coronavirus spike protein restrict viral antigen spread in the central nervous system. *Virology* **2003**, *312*, 369–380. https://doi.org/10.1016/s0042-6822(03)00248-4.
54. Rockx, B.; Donaldson, E.; Frieman, M.; Sheahan, T.; Corti, D.; Lanzavecchia, A.; Baric, R.S. Escape from Human Monoclonal Antibody Neutralization Affects In Vitro and In Vivo Fitness of Severe Acute Respiratory Syndrome Coronavirus. *J. Infect. Dis.* **2010**, *201*, 946–955. https://doi.org/10.1086/651022.
55. De Groot, L.; Piñón, J.D.; Phillips, J.; Lavi, E.; Weiss, S.R. Pathogenesis of Fusion Deficient Recombinant Mouse Hepatitis Viruses. In *The Nidoviruses*, 1st ed.; Lavi E., Weiss S.R., Hingley S.T. Eds.; Springer: Boston, USA, **2001**, 159–162. https://doi.org/10.1007/978-1-4615-1325-4_26.
56. Graham, R.L.; Deming, D.J.; Deming, M.E.; Yount, B.L.; Baric, R.S. Evaluation of a recombination-resistant coronavirus as a broadly applicable, rapidly implementable vaccine platform. *Commun. Biol.* **2018**, *1*, 179. https://doi.org/10.1038/s42003-018-0175-7.
